# Candidate genes with antagonistic roles in stomatal development are associated with population-wide variation in apple

**DOI:** 10.1093/hr/uhag285

**Published:** 2026-07-08

**Authors:** Francesca Zuffa, Michaela Jung, Steven Yates, Carles Quesada-Traver, María-Estefanía López, Giovanni A L Broggini, Andrea Patocchi, Bruno Studer, Graham Dow

**Affiliations:** Molecular Plant Breeding, Institute of Agricultural Sciences, ETH Zurich, Universitaetstrasse 2, Zurich 8092, Switzerland; Laimburg Research Centre, Auer 39040, Italy; Molecular Plant Breeding, Institute of Agricultural Sciences, ETH Zurich, Universitaetstrasse 2, Zurich 8092, Switzerland; Division of Plant Breeding, Agroscope, Mueller-Thurgau-Strasse 29, Waedenswil 8820, Switzerland; Molecular Plant Breeding, Institute of Agricultural Sciences, ETH Zurich, Universitaetstrasse 2, Zurich 8092, Switzerland; Molecular Plant Breeding, Institute of Agricultural Sciences, ETH Zurich, Universitaetstrasse 2, Zurich 8092, Switzerland; Molecular Plant Breeding, Institute of Agricultural Sciences, ETH Zurich, Universitaetstrasse 2, Zurich 8092, Switzerland; Molecular Plant Breeding, Institute of Agricultural Sciences, ETH Zurich, Universitaetstrasse 2, Zurich 8092, Switzerland; Division of Plant Breeding, Agroscope, Mueller-Thurgau-Strasse 29, Waedenswil 8820, Switzerland; Molecular Plant Breeding, Institute of Agricultural Sciences, ETH Zurich, Universitaetstrasse 2, Zurich 8092, Switzerland; Molecular Plant Breeding, Institute of Agricultural Sciences, ETH Zurich, Universitaetstrasse 2, Zurich 8092, Switzerland; Crop Science and Production Systems, NIAB, 93 Lawrence Weaver Road, Cambridge CB3 0LE, UK

## Abstract

A sustainable approach to address climate change and increasing water demand in agriculture is breeding for plant functional traits that conserve water and enhance climate resilience. Stomata regulate plant–water relations and are promising targets for crop improvement. Here, we investigate the variation in stomatal density (SD) in a diverse apple population (*Malus domestica* Borkh.) consisting of 269 accessions. Genome-wide association studies identified robust associations with SD on chromosomes 2, 9, and 10 (classified as SNPs with a *P*-value higher than adjusted Bonferroni threshold of −log10(*P*) > 8.78 that were consistently identified across datasets). On chromosome 9, a candidate gene that negatively regulates stomatal development, EPIDERMAL PATTERNING FACTOR 1 (*EPF1*), was identified inside a genomic region of 241 kb determined by six robust associations. On chromosome 10, a positive regulator candidate gene, EPIDERMAL PATTERNING FACTOR LIKE 9 (*STOMAGEN*), was identified 1680 kb from the robust association. Identification of positive (*STOMAGEN*) and negative (*EPF1*) regulators of SD suggest potential antagonistic roles at the population scale in determining SD. On chromosome 2, a gene co-expression analysis identified a gene cluster containing both *EPF1* and *STOMAGEN* together with a novel candidate gene, CYTOCHROME P450 (*CYP77A4*), that was located 544 kb from the robust association. The percentage of SD phenotypic variance explained by each robust association was between 7% and 10%. These findings provide a foundation for understanding SD variation at the population scale and opportunities to modulate SD by genomics-assisted breeding strategies.

## Introduction

Climate change is predicted to increase the demand for water in agriculture and this will create a major challenge on a global scale. Drought is already the most damaging plant abiotic stress factor, accounting for annual losses of $80 billion USD per year [1]. Despite past genetic improvements enhancing productivity and abiotic stress resilience, yield gains for key staple crops, such as maize (*Zea mays* L.), rice (*Oryza sativa* L.), and wheat (*Triticum aestivum* L.), are now stagnating or diminishing. This suggests the need to shift the selection pressure towards the creation of more resilient crops [2, 3]. Similarly, horticultural breeding programs, including apple (*Malus domestica* Borkh.), have traditionally focused on yield, fruit quality traits, and resistance to biotic stresses because of their economic importance to producers and appeal to consumers [4]. Therefore, climate change demands expanding the focus of fruit breeding programs to include plant functional traits that can enhance resilience to abiotic stresses, such as hotter and drier climatic conditions [5, 6]. Furthermore, abiotic resilience is particularly important in the economics of tree crop production, as orchard establishment requires high investments that are expected to return profits over 15–20 years for a single planting.

One potential adaptation strategy involves the adjustment of stomata, which are microscopic pores on plant surfaces that mediate plant–water relations [7]. While the function of stomata has been studied for centuries [8], the genetic framework that controls stomatal development has only been disclosed by scientists over the past two decades [9]. This discovery provides compelling new targets to improve abiotic stress resilience. The known network of core regulatory genes, first discovered in *Arabidopsis thaliana* L. and since validated across diverse plant species, includes nearly two dozen genes [10, 11]. These core genes exhibit extensive interplay with different signaling networks, endogenous hormones, and also external environmental cues, many of which are still being revealed [10, 11]. The core regulatory genes consist of positively acting transcription factors, cell-surface receptors that interact with a family of small peptide ligands, and a negatively acting mitogen-activated protein kinase cascade [9]. Direct genetic manipulation of stomatal development has revealed many associations between stomatal density (SD), stomatal patterning, and leaf gas exchange in *A. thaliana* L. [12–15]. Stomata also regulate the trade-off between carbon acquisition and water use efficiency (WUE), with their size, distribution, and opening dynamics influencing leaf gas exchange [13]. Stomatal control includes both rapid changes in stomatal aperture through guard cell turgor and long-term modifications in SD [10], influenced by genetic and environmental factors [16].

The impact of SD on plant function has also been validated in important crop species, including a variety of grasses [17–23], tomato (*Solanum lycopersicum* L.; [24, 25]), and grapevine (*Vitis vinifera* L.; [26]). These functional studies have used genetic techniques, such as genome editing and T-DNA insertion approaches to knock out the genes to manipulate SD; however, most studies of diverse apple populations have explored the natural variation of other phenotypic apple traits and not SD [27–33]. When considering dicot species in general, phenotyping stomatal patterning is more complicated than in monocot species where stomata are arranged in rows [25, 34]. This difference in complexity for stomatal phenotyping presents a bottleneck, as it requires considerable investment of resources and time [35]. Nevertheless, deep learning approaches, and more specifically convolutional neural networks (CNNs), have recently become powerful tools for image analysis to enable automated stomatal detection and counting [36].

Phenotyping advancements were complemented by rapid increases in genomic resources. The latter has led to cost-effective, genome-wide information for agricultural crops and native plant species, including apple [37]. In this context, several apple reference genomes have been published since 2010 [38–43]. The development of medium- and high-density single-nucleotide polymorphism (SNP) arrays, such as the Illumina Infinium® 20 K SNP genotyping array [44], followed by the Affymetrix Axiom® Apple 480 K SNP genotyping array [45], and genotyping-by-sequencing methods [30] improved the ability to explore apple genomics. Leveraging similar genotyping tools, genome-wide association studies (GWASs) have emerged as a robust technique for identifying genes that influence quantitative agronomic traits [46, 47]. In apple, GWASs have successfully identified markers associated with phenological, fruit quality, and productivity traits across various apple germplasm collections [27, 29–33, 48]. However, despite the availability of extensive genome-wide information, effectively mining these data for gene discovery remains a challenge. Thus, gene networks were developed to explore correlations in gene expression patterns, offering a systematic approach to understanding the molecular mechanisms underlying targeted biological processes [49]. When combined with known regulatory genes in stomatal development as anchors for network analysis, these methods offer a promising opportunity to explore the genetic basis controlling stomatal traits in apple.

The objectives of this study were: (i) to investigate variation in SD across multiple years based on different stomatal counting datasets; (ii) to calculate the predictive ability and genomic heritability of SD; and (iii) to identify SNPs associated with variation in SD by performing GWAS and identify candidate genes for SD.

## Results

### Counting methods can impact characterization of population-wide stomatal density

Differences in SD distribution were observed between the Manual and the two automated based datasets (Automated, Automated Fullset) (Fig. 1A–C). In 2019, the Manual dataset showed a normal distribution (*P* = 0.11), with a median of 360 stomata per square millimeter. However, in 2020, the Manual data were not normally distributed (*P* = 0.04) and had a median of 325 stomata per square millimeter (Fig. 1A). In contrast, both datasets based on the Automated (*P* = 0.79 in 2019, *P* = 0.45 in 2020), and the Automated Fullset (*P* = 0.81 in 2019, *P* = 0.45 in 2020, *P* = 0.40 in 2021) were normally distributed in all years. Automated had a median SD of 362 stomata per square millimeter and 339 stomata per square millimeter in 2019 and 2020, respectively (Fig. 1B). Similarly, Automated Fullset had median SD of 361 stomata per square millimeter (2019), 340 stomata per square millimeter (2020), and 339 stomata per square millimeter (2021, Fig. 1C). The SD population median in 2019 differed significantly from both 2020 and 2021 for all three datasets (*P* < 0.001) (Fig. 1A–C). The median SD in 2020 from Manual was also significantly different from the 2020 mean in Automated and Automated Fullset *(P* < 0.05). The Manual SD distribution had a lower peak and wider shoulders relative to the Automated distribution. This difference was reflected in the regression analysis between Manual and Automated, whereby deviation from the 1:1 line was greater at high and low SD for Manual (Fig. 1D). A strong correlation was still observed between Manual and Automated for each microscope image (*n_image_* = 6234) with Pearson correlation coefficient (*r)* = 0.89, Root Mean Square Error (*RMSE*) = 31.54 and the leaf average (2–3 images per leaf, *n_leaf_* = 2319) with *r* = 0.93 and *RMSE* = 28.65 (Fig. 1D), whereby *RMSE* indicates the average deviation between manual counts and the model prediction in stomata per square millimeter. The stomatal detection by the automated algorithm is shown in Fig. 1E and F, as demonstrated by the annotated microscope images.

**Figure 1:**
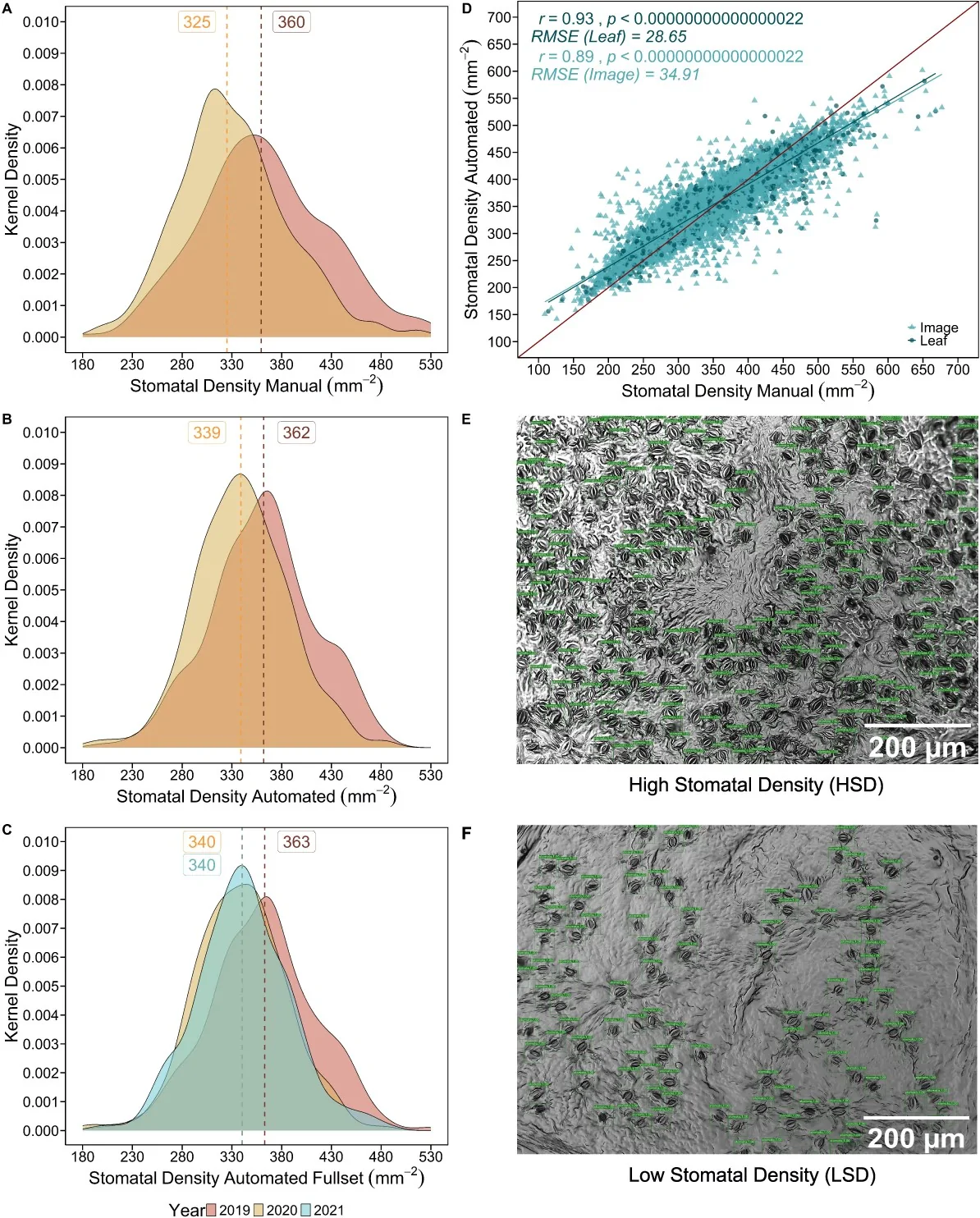
Stomatal density (SD) variation in the apple REFPOP with manual and automated counting. **(**A) Manual counting SD distribution curves of 269 accessions in the years 2019 and 2020 (*n_image_* = 6234; *n_leaf_* = 2319). (B) Automated counting SD distribution curves of the same set of images as in (A) (*n_image_* = 6234; *n_leaf_* = 2319). (C) Automated Fullset counting SD distribution curves of 269 accessions in the years 2019, 2020, and 2021 (*n_image_* = 12 612; *n_leaf_* = 3633). The median SD was calculated for each year in all datasets and is represented by a vertical dashed line and boxed value. (D) Relationship between the manually and automatically counted data from (A) and (B). Root Mean Square Mean Error (*RMSE*) are indicated at the image (*n_image_* = 6234; cyan) and leaf level (*n_leaf_* = 2319; blue). The 1:1 line is shown in red. Abaxial epidermal images of (E) High Stomatal Density (HSD) and (F) Low Stomatal Density (LSD) accessions with deep learning segmentation annotation.

### Moderately high genomic heritability and predictive ability for stomatal density in apple

The phenotypic variance decomposition revealed, on average across all the three datasets, that 39% of SD phenotypic variance was explained by genotype, 22% by the genotype by year by leaf interaction, and 11% by the genotype by year interaction, and only 3% were explained by the year alone (Fig. 2A). When comparing percentages across datasets, Manual had the highest percentage of SD variance explained by the genotype (42%), the lowest percentage explained by the interaction between the genotype by year by leaf (21%), and the lowest residuals (22%). In contrast, Automated Fullset had the highest relative percentage of SD variance explained by the leaf (25%), while Automated had the highest relative effect of genotype by year interaction (12%) (Fig. 2A).

**Figure 2:**
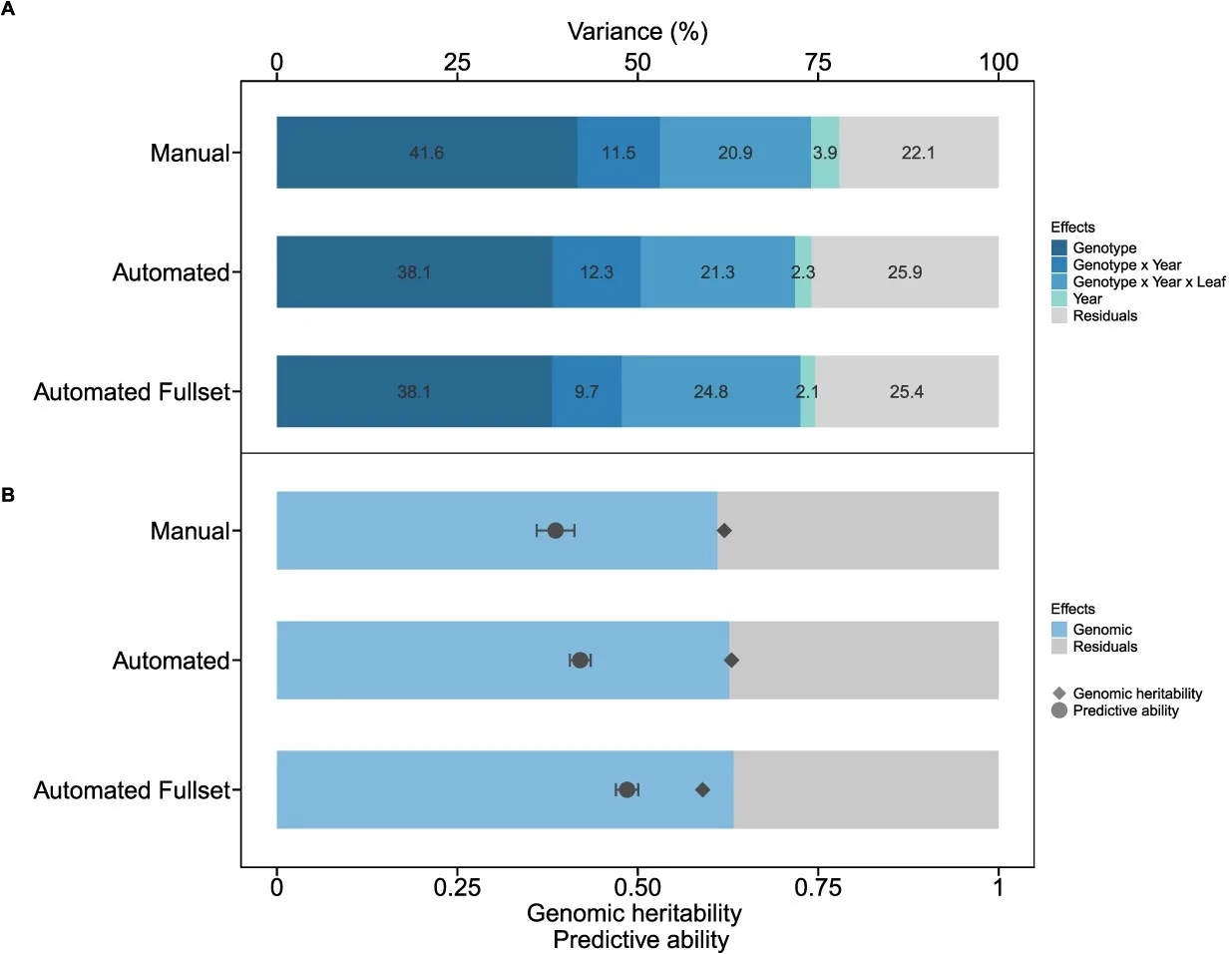
Stomatal density (SD) phenotypic variance decomposition, genomic heritability, and predictive ability. Stacked bar plots with A) the percentage of SD phenotypic variance explained by the fixed effect (year) and random effects (genotype, genotype by year interaction, genotype by year by leaf interaction, and residuals) as calculated from the linear mixed model fit for each SD dataset [Manual (*n_image_* = 6234), Automated (*n_image_* = 6234), and Automated Fullset (*n_image_* = 12612)]; B) the percentage of SD genomic variance as explained by SNP markers and the residual variance not explained by SNP markers calculated for each SD dataset [Manual (*n_image_* = 6234), Automated (*n_image_* = 6234), and Automated Fullset (*n_image_* = 12612)]. Genomic heritability (black diamond) and average predictive ability (black points with error bars) are included for each dataset. The error bars correspond to standard deviation around the average predictive ability.

The genomic prediction model resulted in greater differences for SD-predictive ability among the three datasets, while genomic heritability (${h}^2$) was more consistent. Across the three datasets, ${h}^2$ was 0.62 for Manual, 0.63 for Automated, and 0.59 for Automated Fullset (Fig. 2B), with an average ${h}^2$ of 0.61. The predictive ability was 0.39 for Manual, 0.42 for Automated, and 0.49 for Automated Fullset, with an average of 0.43. Furthermore, the genomic variance was 61% for the Manual and 63% for both the Automated and Automated Fullset (Fig. 2B).

### Association of genomic loci with stomatal density on chromosomes 2, 9, and 10

SNP markers significantly associated with SD were identified on chr 1, 2, 3, 4, 5, 9, 10, 11, 12, 15, and 16 (Fig. 3, Table S1 for the list of −log_10_ (*P*-value) and ${R}^2$). The Automated Fullset showed lower *p*-values compared to the other two datasets (Manual, Automated). Specifically, on chr 2, 9, and 10, BLINK consistently found significant marker–trait associations across all three datasets with −log_10_(*P*-value) above the adjusted Bonferroni-corrected significance threshold with many SNPs in those regions showing robust association (Fig. 3, Table S1 for the list of −log_10_ (*P*-value) and ${R}^2$). A robust association is defined here as being characterized by high ${R}^2$ values, significant low *P*-values, and consistently identified for Manual and both automated datasets (Automated and Automated Fullset) using BLINK. The model GEMMA was also applied using the resampling strategy, resulting in additional 100 GWASs for each dataset (Fig. 3; Table S1). GEMMA found significant associations based on the standard Bonferroni-corrected significance threshold on chr 3, 4, 6, and 7 but none based on the adjusted Bonferroni-corrected significance threshold (Fig. 3).

**Figure 3:**
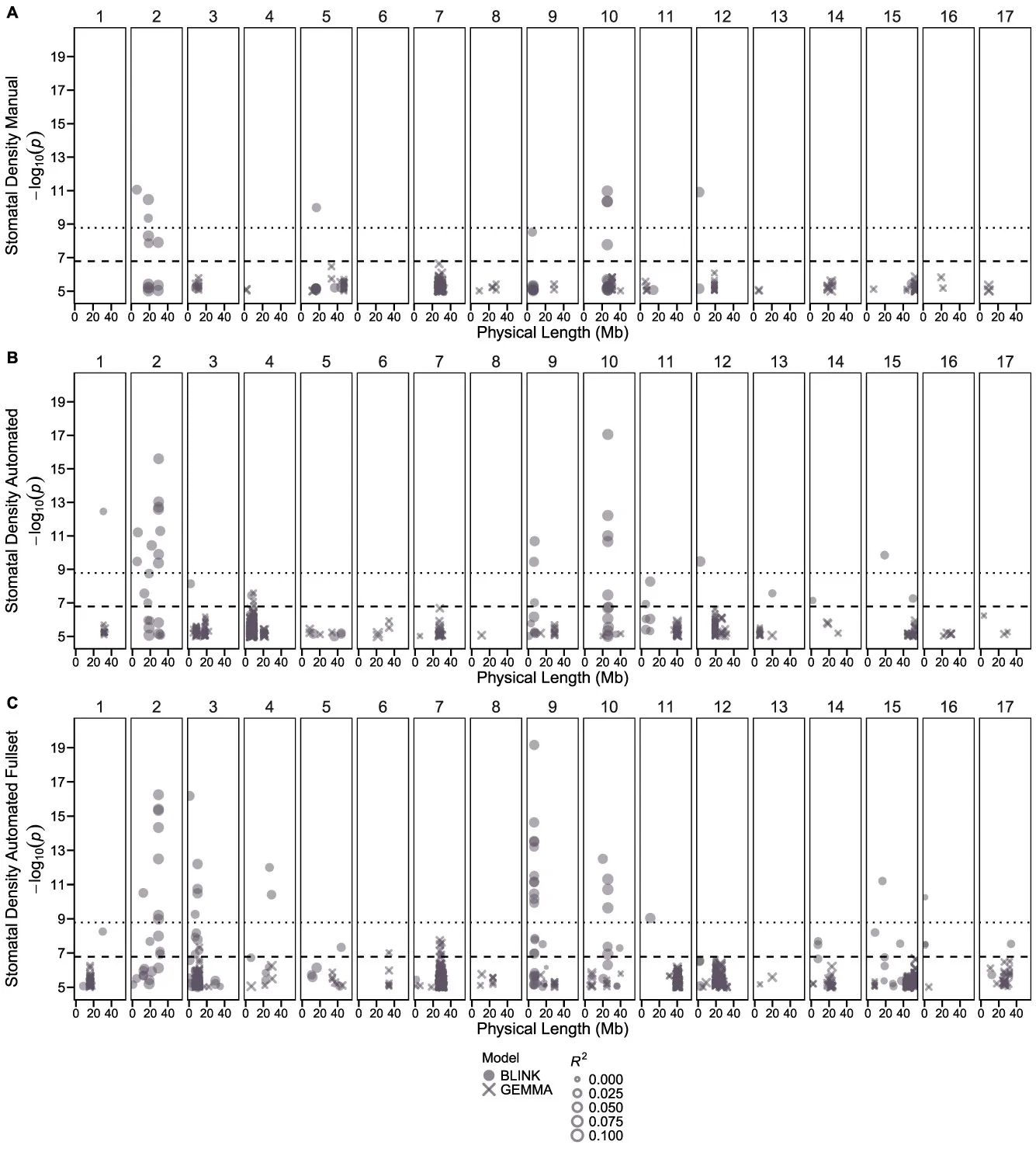
Stomatal density (SD) genome-wide association study (GWAS) for each individual dataset. Manhattan plot with −log_10_-transformed *P*-values obtained by using a resampling GWAS for each SD dataset: (A) Manual (*n_image_* = 6234); (B) Automated (*n_image_* = 6234); (C) Automated Fullset (*n_image_* = 12612); with two different models: BLINK (circles), GEMMA (crosses). The *x*-axis indicates the physical SNP position along each chromosome, and the *y*-axis indicates the SNP -log_10_ (*P*-value) above 5. The dashed horizontal line indicates the standard Bonferroni threshold for significant SNP associations within each GWAS and the dotted line indicates the adjusted Bonferroni threshold, with the significance level adjusted for the number of GWASs performed for each dataset. Point sizes indicate the coefficient of determination (${R}^2$) for each SNP (scale only shown for circles).

For further analysis, we focused on the most robust associations found with BLINK based on the adjusted Bonferroni-corrected significance threshold on chr 9 (AX-115328310, AX-115358303, AX-115502470, AX-115502472, AX-115502475, AX-115603698, Fig. 4A), chr 10 (AX-115275897, AX-115603212, Fig. 5A), and chr 2 (AX-115282570, AX-115201850, AX-115314030, AX-115314237, AX-115303382, AX-115254027, AX-115539968, AX-115495218, AX-115413588, Fig. 6A). Three SNPs on chr 9 (AX-115502472, AX-115502475, AX-115603698) each accounted for ∼9% of the phenotypic variance, as indicated by the ${R}^2$, while the remaining SNPs (AX-115328310, AX-115358303, AX-115502470) contributed at most ∼7% each (Fig. 4A; Table S1). The average difference in SD between the homozygous allelic combinations across all six SNPs was 37 stomata per square millimeter (Fig. 4B). Similarly, on chr 10, AX-115603212 accounted for ∼10% of the phenotypic variance, whereas AX-115275897 accounted for ∼7% (Fig. 5A; Table S1). The average difference in SD between the homozygous allelic combinations across both SNPs was 44 stomata per square millimeter (Fig. 5B). On chr 2, AX-115495218, AX-115539968, and AX-115413588 accounted for ∼10%, ∼9%, and ∼7% of the phenotypic variance, respectively (Fig. 6A; Table S1). The average difference in SD between the homozygous allelic combinations across all three SNPs was 37 stomata per square millimeter (Fig. 6B).

**Figure 4:**
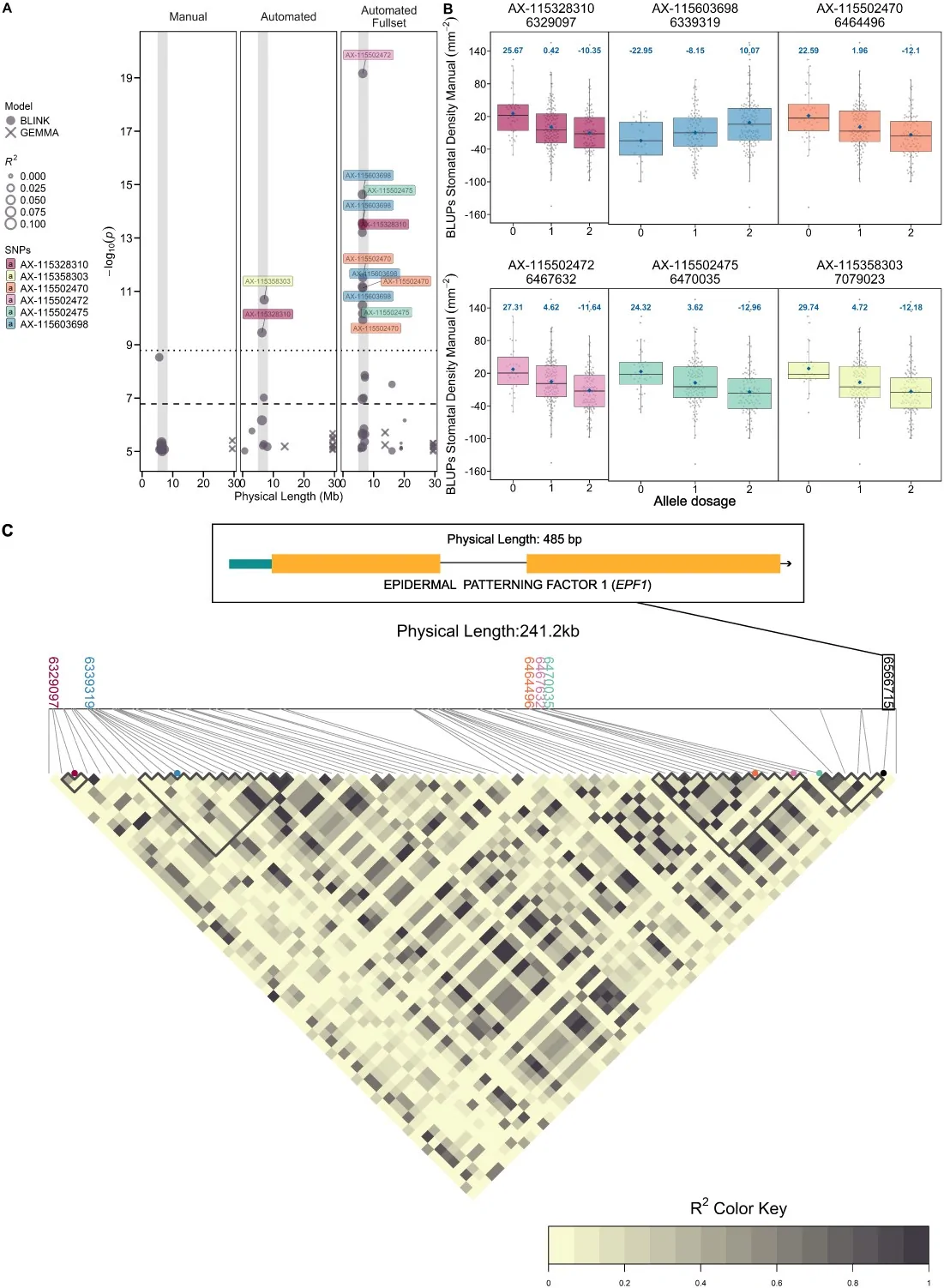
SNP associations with stomatal density (SD) on chromosome 9 and identification of the putative candidate gene EPIDERMAL PATTERNING FACTOR 1 (*EPF1)*. (A) Manhattan plot on for chromosome 9 with the robust associations labelled using their SNP name. The dashed horizontal line indicates the standard Bonferroni threshold for significant SNP associations within each GWAS. The dotted line indicates the adjusted Bonferroni threshold accounting for the number of GWAS performed for each dataset. (B) Boxplots of allelic effects for each robust association above the dotted line indicating the adjusted Bonferroni threshold. Individual Manual SD data points and mean value (blue diamond, blue number) are shown. The *x*-axis indicates allelic dosage (0 copies of the major allele, 1 copy of major allele, 2 copies of major allele, with the major allele representing the most frequent allele in a biallelic SNP) and the *y*-axis indicates the change in SD using values extracted from the linear mixed model (Best Linear Unbiased Predictors, BLUPs) arising from allelic combinations. (C) LD heatmap of 241.2 kb region on chromosome 9 showing physical locations of each robust association (AX-115328310, AX-115603698, AX-11552470, AX-115502472, AX-115502475) above the adjusted Bonferroni threshold, their haploblocks (black triangles), and the physical location of EPIDERMAL PATTERNING FACTOR 1 *(EPF1)*. Schematic illustration of the predicted gene structure for *EPF1*, including the promoter region (turquoise), coding sequences (orange), and intron (black line).

**Figure 5:**
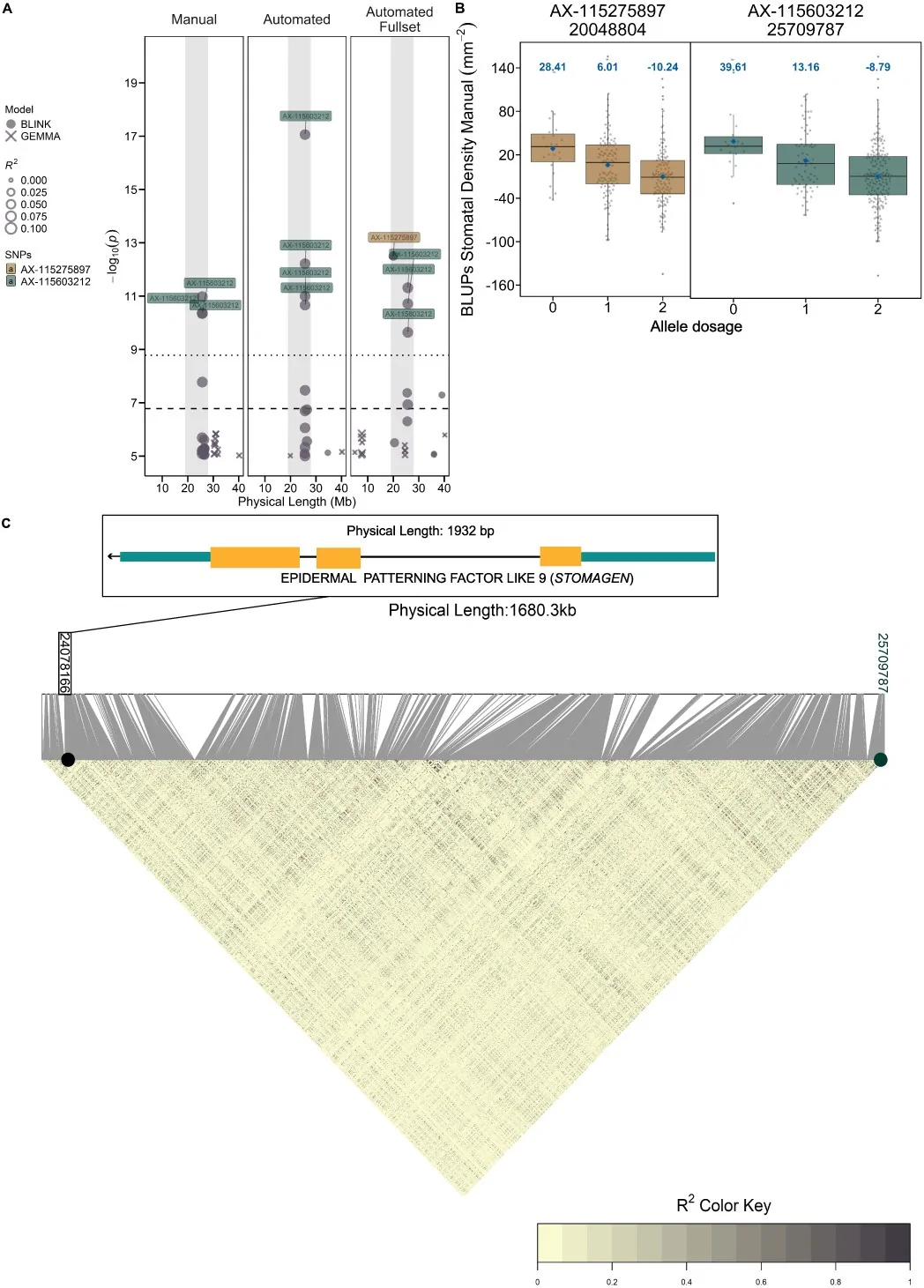
SNP associations with stomatal density (SD) on chromosome 10 and identification of the putative candidate gene EPIDERMAL PATTERNING FACTOR LIKE 9 (*STOMAGEN)*. (A) Manhattan plot on chromosome 10 with the robust associations labeled using their SNP name. The dashed horizontal line indicates the adjusted Bonferroni threshold accounting for the number of GWAS performed for each dataset. (B) Boxplots of allelic effects for each SNP above the dotted line indicating the adjusted Bonferroni threshold accounting for the number of GWAS. Individual Manual SD data points and mean value (blue diamond) are shown. *X*-axis indicates allelic dosage (0 copies of the major allele, 1 copy of major allele, 2 copies of major allele, with the major allele representing the most frequent allele in a biallelic SNP) and *y*-axis indicates the change in SD using values extracted from the linear mixed model (Best Linear Unbiased Predictors, BLUPs) arising from allelic combinations. (C) LD heatmap of 1680.3 kb region on chromosome 10 showing physical locations of the robust association (AX-115603212) above the adjusted Bonferroni threshold and the physical location of EPIDERMAL PATTERNING FACTOR LIKE 9 *(STOMAGEN)*. Schematic illustration of the predicted gene structure for *STOMAGEN*, including the promoter regions (turquoise), coding sequences (orange), and introns (black line).

**Figure 6:**
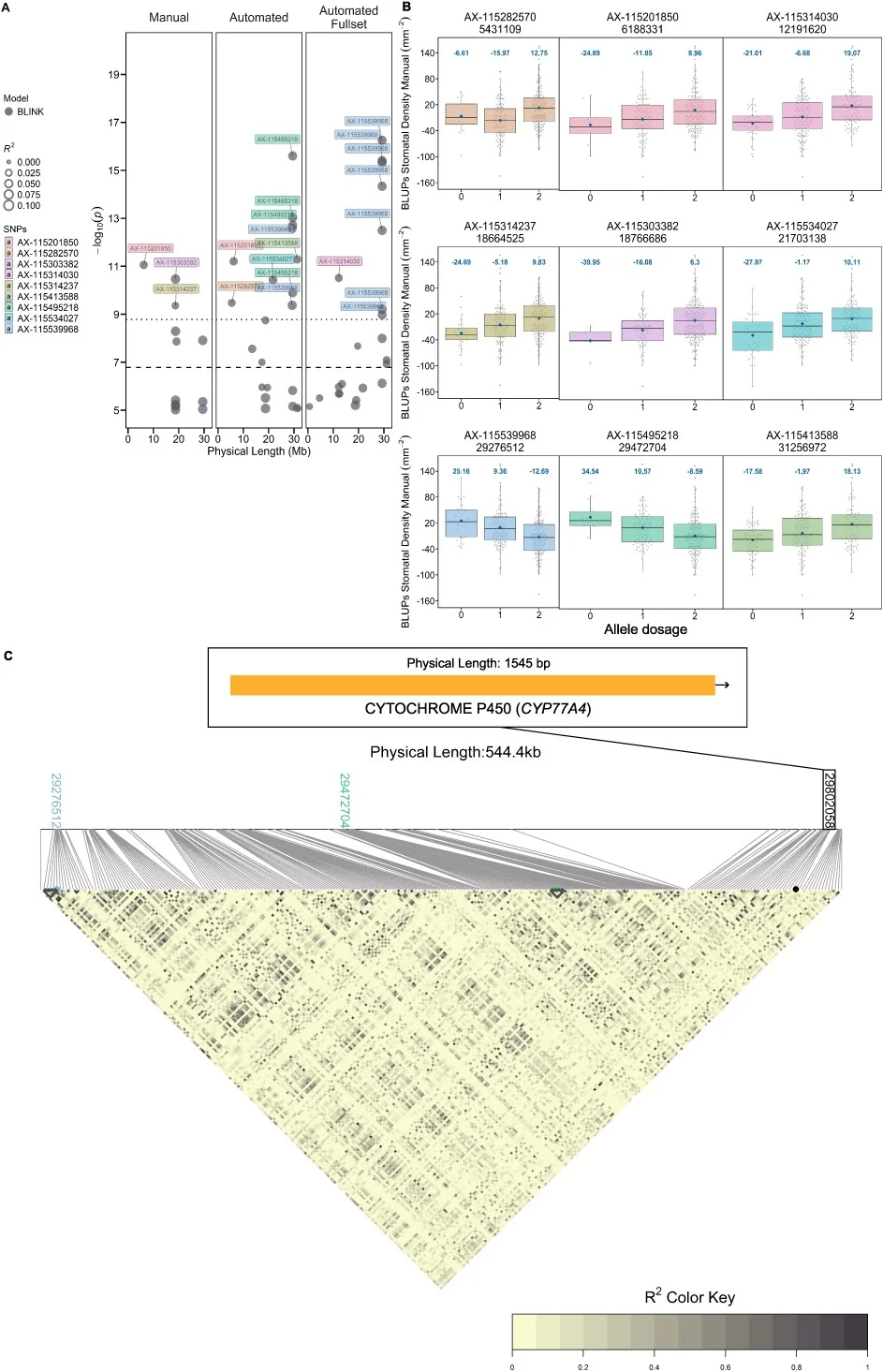
SNP associations with stomatal density (SD) on chromosome 2 and identification of the putative candidate gene CYTHOCROME P450 (*CYP77A4*). (A) Manhattan plot on chromosome 2 with the robust associations labelled using their SNP name. The dashed horizontal line indicates the adjusted Bonferroni threshold accounting for the number of GWAS performed for each dataset. (B) Boxplots of allelic effects for each SNP above the dotted line indicating the adjusted Bonferroni threshold accounting for the number of GWAS. Individual Manual SD data points and mean value (blue diamond) are shown. *X*-axis indicates allelic dosage (0 copies of the major allele, 1 copy of major allele, 2 copies of major allele, with the major allele representing the most frequent allele in a biallelic SNP), and *y*-axis indicates the change in SD using values extracted from the linear mixed model (Best Linear Unbiased Predictors, BLUPs) arising from allelic combinations. (C) LD heatmap of 544.4 kb region on chromosome 2 showing physical locations of robust association (AX-115539968, AX-115495218) above the adjusted Bonferroni threshold, their haploblocks (black triangles), and the physical location of CYTHOCROME P450 (*CYP77A4*). Schematic illustration of the predicted gene structure for *CYP77A4*, including coding sequences (orange), and intron (black line).

### Putative regulators of stomatal development are associated with variation in stomatal density

Candidate genes associated with SD variation in apple were identified using the robust associations found with BLINK across the three datasets (Fig. 3). On chr 9, the six SNPs spanned over four haploblocks within a genomic region of ~241 kb in total physical length (Fig. 4C). Within the haploblock identified by SNP AX-115502475, the putative candidate gene EPIDERMAL PATTERNING FACTOR 1 *(EPF1;* MD09G1089600) was found (Fig. 4C). On chr 10, the significantly associated SNP AX-115603212 with the lowest *P*-value identified a haploblock located ~1680 kb from the putative candidate gene EPIDERMAL PATTERNING FACTOR LIKE 9 *(STOMAGEN;* MD10G1153200) (Fig. 5C). Additionally, the significantly associated SNP AX-115603212 was located 1680 kb from *STOMAGEN*, and another SNP, AX-115275897, was located 4029 kb away, with the putative gene *STOMAGEN* positioned between the two SNPs. When the two SNPs (AX-115502475, AX-115603212) that were used to identify the candidate genes (*EPF1* and *STOMAGEN*) were put into a multilocus genotype (Fig. S1A), they explained 13%–15% of the phenotypic variance in SD, depending on the dataset (Fig. S1A). The maximum average difference in SD between allelic combinations at these loci was 79 stomata per square millimeter (Fig. S1B). Combinations of *STOMAGEN* alleles with dosage 1 or 2 at AX-115603212 and *EPF1* alleles with dosage 2 at AX-115502475 showed the most negative effects on SD (Fig. S1B; multilocus genotypes 22 and 21). *STOMAGEN* allele dosage 0 at AX-115603212 showed positive effects on SD when combined with any *EPF1* allele dosage (0, 1, or 2) at AX-115502475 (Fig. S1B; multilocus genotypes 20, 10, and 00).

Inspecting the genomic region of the nine robust associations on chr 2 associated with SD, no candidate gene with an obvious annotation related to SD was found. However, using the weighted gene co-expression network analysis (WGCNA), *EPF1* and *STOMAGEN* were confirmed as central players in stomatal regulation (Fig. 7). These candidate genes (*EPF1*, *STOMAGEN)* were identified within network 11 of the 69 networks derived from the WGCNA (Table S3). This network comprised 647 nodes (genes) and 4289 edges (connections) (Table S3). A heatmap of differential tissue expression profiles for genes in network 11 predominantly showed differential expression in leaf tissues (Fig. S3). Functional enrichment analysis was also conducted to explore the biological roles of the genes in network 11 (Fig. S4; Table S4). Network 11 was significantly enriched (*P_FDR_* < 0.05) for genes involved in stomatal lineage progression (GO:0010440, 6 genes), stomatal complex morphogenesis (GO:001010, 23 genes), and stomatal complex development (GO:0010374, 33 genes) (Fig. S3; Table S4). Both candidate genes, *EPF1* (MD09G1089600) and *STOMAGEN* (MD10G1153200), were included in the pathway for stomatal complex development derived from the functional enrichment analysis (Fig. S3). Other known regulatory genes in stomatal development were also included in network 11, including putative *EPF2* (MD00G1078200), *FAMA* (MD04G1006400) and *SCREAM* (MD14G1148600, MD06G1132200), and a potential paralog of *STOMAGEN* (MD05G1163400) (Fig. 7; Table S3). When considering only the first neighboring genes of our candidates (*STOMAGEN, EPF1*) and the other known stomatal genes (*EPF2*, *FAMA*, *SCREAM*, paralog *STOMAGEN*), a total of 79 connected genes were significantly correlated to each other (*P* < 0.001, Fig. 7). This network was used to identify a novel putative candidate gene, CYTOCHROME P450 (*CYP77A4*) on chr 2, which was located ~544 kb from two robust associations (AX-115539968, AX-115495218, Fig. 6C). However, including the closest SNP to *CYP77A4* (AX-115495218) in the multilocus genotype with SNPs from chr 9 (AX-115502475) and chr 10 (AX-115603212) did not increase the amount of phenotypic variance explained compared to the multilocus genotype with only SNPs from chr 9 (AX-115502475) and chr 10 (AX-115603212) (13%–14%, Figs S1A vs S4A).

**Figure 7:**
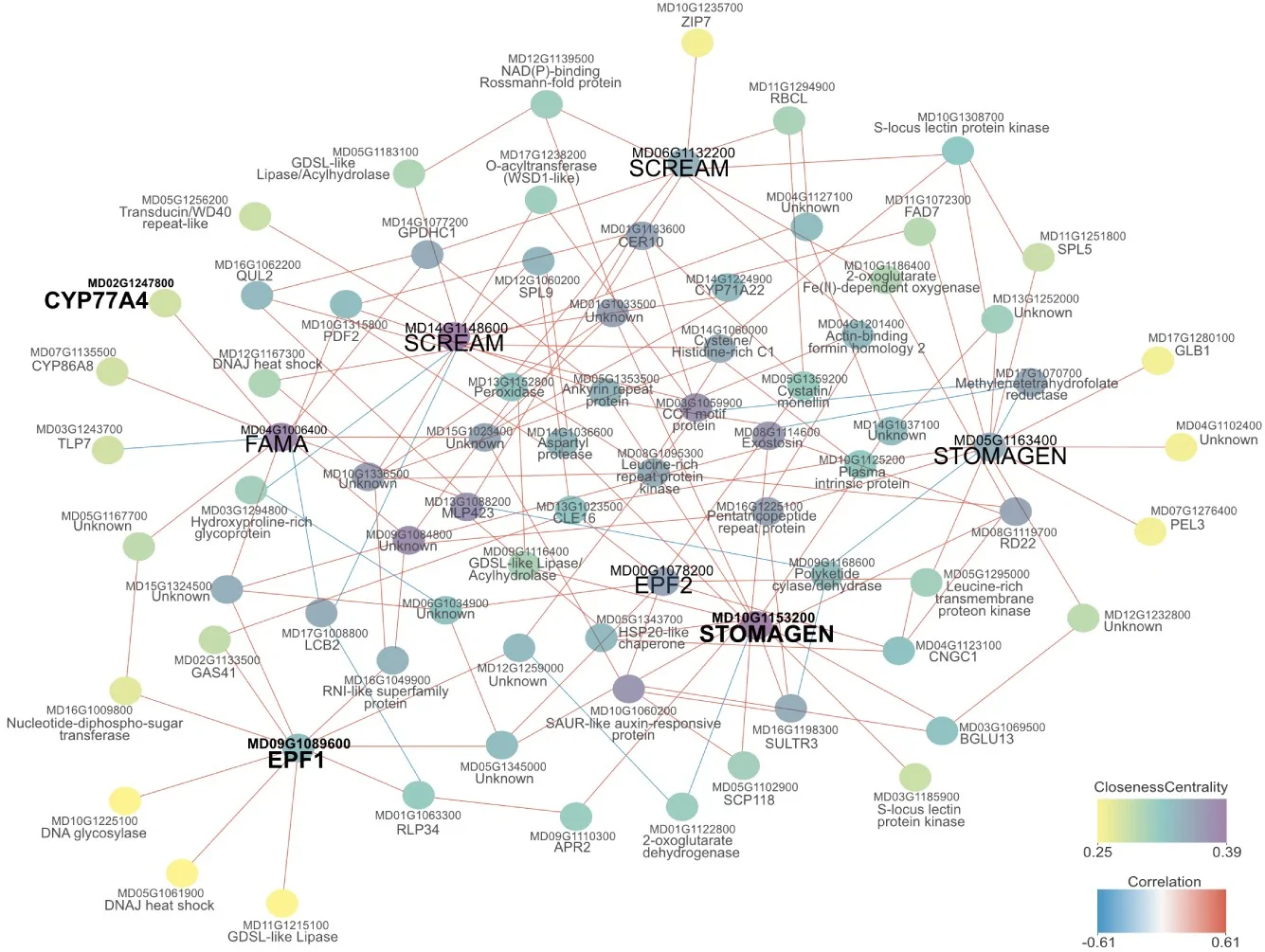
Weighted gene co-expression network analysis of known and putative candidate genes involved in stomatal development. First-neighboring genes and only significantly co-expressed genes are shown (*P* < 0.001). The network displays first-neighboring genes that are significantly co-expressed (*P* < 0.001), with *P*-values adjusted using Bonferroni correction. Each node represents an annotated gene ID (using the reference genome GDDH13 v1.1), along with the common name of the gene, when known. Genes identified by our GWAS in apple (large font, bold, black colored) and those known to be associated with stomatal development in other plants (large font, black colored) are highlighted. Highlighted in the network are genes identified through GWAS in apple, shown in large, bold black font, and those known to be associated with stomatal development in other plants, shown in large black font. Other genes are labeled gray. Node color indicates the connectedness and centrality of each gene within the network, ranging from 0.25 to 0.39. Yellow nodes represent genes with fewer connections and less centrality, while purple nodes indicate genes with more connections and greater centrality. Lines between nodes show the correlation of gene expression: a positive (red-colored) and negative (blue-colored) correlations of gene expression.

### Different apple genomes as a resource to examine structural variation characteristics in control elements of the candidate genes

The whole-genome sequences of 14 accessions were assembled and annotated to explore gene structure and to analyze potential structural variation around the identified candidate genes (*EPF1*, *STOMAGEN*, and *CYP77A4*) (Fig. 8). These three candidate genes (*EPF1*, *STOMAGEN*, and *CYP77A4*) were found to be highly conserved across all the analyzed genomes (Fig. 8). Specifically, *STOMAGEN* was located on the reverse DNA strand, whereas *EPF1* and *CYP77A4* were found on the forward strand. Analysis of genomic regions in proximity of *EPF1* revealed some structural variation in six accessions (‘Esopus Spitzenburg’, ‘Priscilla’, ‘McIntosh’, ‘Prima’, ‘Jonathan’, ‘Edward VII’), where at least one haplotype differed from the others. These accessions exhibited the insertion of two transposable elements (TEs) of the *RTE-BovB* downstream of *EPF1*, as well as two *Copia* TEs upstream. Additionally, across all analyzed genomes, *EPF1* was found to be 1172 bp from a gene annotated as MD09G1089700, a member of the *ELMO/CED-12* TE family (Fig. 8A).

**Figure 8:**
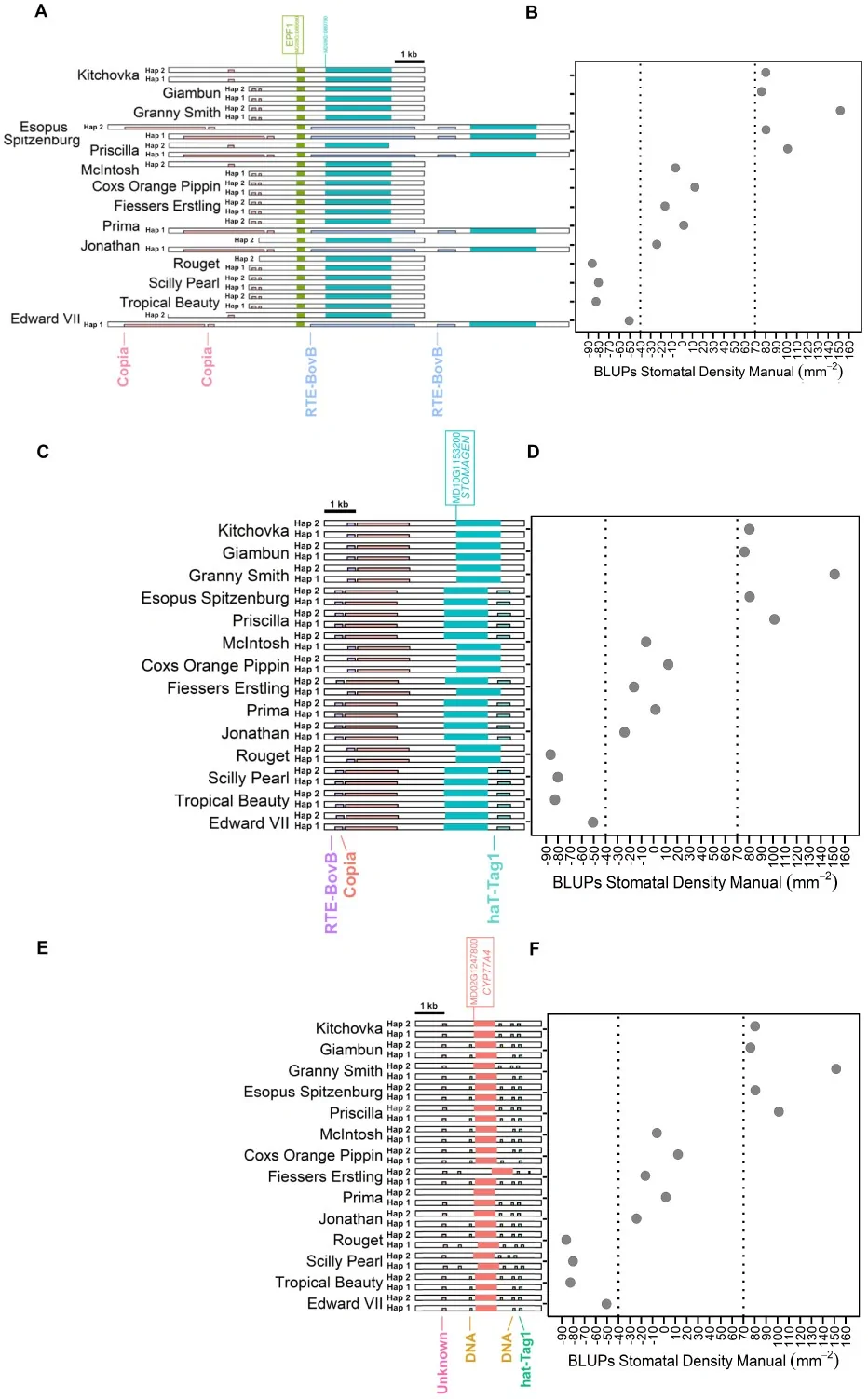
Different accession-specific genomes as a resource to examine structural variation near the putative genes: *STOMAGEN* (chromosome 10), *EPF*1 (chromosome 9), and *CYP77A4* (chromosome 2). Comparison of 14 apple genotypes with different SD in the genomic region around the putative gene *EPF1* (A), *STOMAGEN* (C), and *CYP77A4* (E). Each genome is shown with two haplotypes, representing a pair of chromosomes. The green rectangle indicates *EPF1*, annotated as MD09G1089600 in the reference genome (GDDH13) (A). The cyan rectangle indicates *STOMAGEN*, annotated as MD10G1153200 in the reference genome (GDDH13) (C). The red rectangle indicates *CYP77A4* annotated as MD02G1247800 in the reference genome (GDDH13) (E). The colored rectangles with black frames indicate different transposable elements (TEs) (A, C, E). (B, D, F) Scatterplots with phenotypic Best Linear Unbiased Predictor (BLUP) values for Manually counted stomatal density (SD) for each of the genotypes. Dotted vertical lines identify the phenotypic classification of genotypes with high stomatal density (HSD, above 70) and low stomatal density (LSD, below −40).

In the genomic region surrounding *STOMAGEN*, all analyzed genomes contained downstream insertions of *Copia* and *RTE-BovB* TEs. Upstream of the gene, a hAT transposon (*hat-Tag1*) was identified in both haplotypes of seven accessions (‘Esopus Spitzenburg’, ‘Priscilla’, ‘Prima’, ‘Jonathan’, ‘Scilly Pearl’, ‘Tropical Beauty’, ‘Edward VII’), whereas five accessions (‘Kitchovka’, ‘Giambun’, ‘Granny Smith’, ‘Cox Orange Pippin’, ‘Rouget’) exhibited a deletion of this element in the putative promoter region (Fig. 8C). Despite this observed variation, the presence or absence of *hat-Tag1* did not correlate with SD phenotypic differences (Fig. 8D).

For *CYP77A4*, structural analysis showed that all genomes contained a hAT transposon (*hat-Tag1*) insertion downstream of the gene, along with an unidentified TE in the upstream of the gene (Fig. 8E). Another DNA TE was identified both upstream and downstream of the gene (Fig. 8E). Similar to the other candidate genes, no clear segregation of these structural variants was observed between SD groups (Fig. 8F). Overall, while structural variations were detected near the candidate genes, none exhibited a strong association with SD.

## Discussion

In our study, a comparison of model BLINK with the model GEMMA showed that BLINK consistently found robust associations across multiple chromosomes, while GEMMA identified fewer significant regions (Fig. 3). This corroborates previous research showing that multilocus GWAS models, such as BLINK, outperform single-locus GWAS methods, like GEMMA [27, 50, 51]. The single-locus GWAS approach GEMMA has known limitations for quantitative traits influenced by multiple genomic regions [52], largely because of the stringent multiple-testing corrections applied to *P*-value thresholds that can cause true associations to be missed [51]. In contrast, BLINK overcomes these issues by incorporating genetic information from multiple loci simultaneously while also accounting for confounding effects of kinship and population structure with improved computational speed and power to detect significant associations [50]. Although BLINK identified robust associations, GEMMA, as a single-locus GWAS method, produced distinct results. Including GEMMA comparisons help to illustrate how different statistical approaches can capture different signals, particularly for quantitative traits controlled by multiple genomic regions, like SD. This comparison highlights the advantages of multilocus methods like BLINK while showing the potential limitations of relying only on single-locus models. Furthermore, the REFPOP population exhibits minimal population structure [53], yet both GEMMA and BLINK account for kinship and relatedness, emphasizing that differences in results reflect methodological differences rather than population structure. Together with *de novo* GWAS, this multimodel approach strengthens confidence in the SD associations identified by BLINK and provides a more comprehensive understanding of the SD genetic architecture.

A comparison of GWAS results from the Manual and Automated datasets revealed the same significantly associated genetic loci at the identical physical positions along the chromosome, with the Automated datasets displaying lower *P*-values (Fig. 3; Table S1). However, despite the Automated datasets resulting in lower *p*-values in the GWAS and significantly reducing the time required for phenotyping SD, they did not fully capture the population’s variability or the nuances of SD within the population (Fig. 1). The Automated Fullset showed lower *P*-values compared to the other two datasets (Manual, Automated) due to the larger dataset size in terms of the number of evaluated years and total replicates, which likely contributed to a more precise estimation of Best Linear Unbiased Predictors (BLUPs) and increased the ability to detect marker–trait associations. Therefore, while Automated approaches offer improved efficiency and statistical power, comparison with the Manual dataset remained indispensable. Furthermore, a potential limitation of this study is that SD phenotyping was conducted on a single biological replicate each year, which may not capture spatial variation in the orchard nor intragenotype variation. As a result, tree-to-tree variation was not explicitly included in the models used to estimate genetic BLUPs or to calculate heritability. Incorporating multiple tree replicates per genotype would have likely captured an additional source of random variation, similar to that observed among leaves (Fig. 2). The robustness of detected marker–trait associations would therefore benefit from validation in other apple populations or breeding pools. Nonetheless, related studies in the apple REFPOP have shown low phenotypic plasticity in SD across tree replicates and geographic locations [55]. The latter study utilized a replicated multienvironment dataset and produced a Pearson correlation coefficient of 0.93 (*P* < 0.001) at the genotype scale (SD of 2–4 trees per genotype per location; *n* = 25 genotypes and 4 locations). This observed stability of SD across biological replicates provides further confidence in the GWAS results presented here and the applicability toward breeding.

Our study also revealed the genomic architecture underlying SD in a genetically diverse apple population. By integrating phenotypic and genotypic data, robust associations on chromosomes 2, 9, and 10 identified putative candidate genes responsible for SD regulation (Fig. 3), suggesting a polygenic genetic architecture of the SD trait in apple. Polygenic control of SD has been well documented in other crops, such as sorghum [17], rice [19, 56], wheat [57], maize [58], and tomato [25]. Furthermore, this study is the first to identify a pair of candidate genes in the *EPF* family via GWAS: *EPF1* on chr 9 (Fig. 4) and *STOMAGEN* on chr 10 (Fig. 5). These putative orthologs have previously been confirmed to control SD in the plant model *A. thaliana* L. [9, 12, 15] and nonmodel plant systems, like barley (*Hordeum vulgare* L.) [21], rice [18, 59, 60], wheat [20], grapevine [26], and sorghum [61], via direct genetic manipulations. Canonically, *EPF1* encodes a small peptide ligand that negatively regulates stomatal development and reduces SD, while *STOMAGEN* encodes a related small peptide ligand that positively regulates stomatal development to increase SD [9]. The developmental hypothesis is that members of the *EPF* family compete for binding sites in the cell-surface receptors of the stomatal lineage (*TMM*, *ER*, *ERL1*, and *ERL2*) to adjust the final number of stomata produced by the leaf [14, 62]. Our results provide indirect evidence that competitive binding of *EPF* family members might also control variation in SD at the population scale in the apple REFPOP. The additive effects of different allele combinations in the multilocus genotype with *EPF1* and *STOMAGEN* loci offer support to this finding (Fig. S2). This is the first study to identify potential interactive gene effects of known regulators on SD. These allelic interactions provide a model for understanding cellular mechanisms at the population scale in nonmodel plant species, and these observations highlight the importance of specific allele combinations for molecular breeding, which is novel in comparison to previously published GWAS results for SD.

Beyond the core regulatory genes acting in the stomatal lineage, numerous downstream factors regulate developmental outcomes, including cell division, cell growth, and differentiation. We found that leaf and stomatal development genes are closely associated in co-expression network (Fig. 7) and functional enrichment analysis (Fig. S3) in apple. The gene co-expression network analysis was instrumental in identifying a potentially new actor in the stomatal lineage, CYTOCHROME P450 (*CYP77A4*), which is located on chr 2 alongside SNPs that were significantly associated with SD (Fig. 7). Cytochrome P450s are a large superfamily of enzymatic proteins that play many roles in plant growth and development, including embryogenesis, germination, and organ patterning [63]. More specifically, *CYP77A4* is proposed to determine the transient location of PIN-FORMED 1 (*PIN1*) that establishes auxin-mediated cell polarity in developing plant embryos [64]. PIN-mediated auxin concentration and stomatal-lineage specific protein polarization are both requisite components in the development of stomata [65, 66]. Given the expression of putative *CYP77A4* in apple leaf tissues with known stomatal regulators, and its role in other species, *CYP77A4* may functionally contribute to the establishment of cell polarity in the stomatal lineage in apple. The integration of multiple approaches, such as *de novo* GWAS, co-expression analysis, and WGS provides complementary insights that would not be captured by any single method alone. GWAS identifies associated loci and controls for any confounding effects of population structure, co-expression analysis prioritizes candidate genes based on functional networks, and genome assemblies allow precise gene mapping and validation across different individuals. Together, these methods have been used here to increase confidence in candidate gene identification by combining statistical and genomic evidence.

The combined impact of the robust SNPs, which identified the putative candidate genes, is also important for evaluating their interactive effects on SD phenotypic variance. The multilocus SNP genotypes accounted for a maximum of 15% of SD variation (Figs S1A and S4A). Polygenic traits often involve other molecular mechanisms in determining phenotypes, such as incremental allelic dosage effects, gene interaction networks, positional effects, and structural variation [67]. Prior research in *Malus* species has demonstrated the critical role of TE variation, particularly in the genomic regions surrounding the v-myb avian myeloblastosis viral oncogene homolog (*MYB*) genes, in driving allele-specific expression. These TE insertions have been found to be responsible for anthocyanin accumulation in apple petals for flower pigmentation [68] as well as red fruit skin [43] and red fruit flesh [69]. The role of structural variation in SD regulation may be further investigated by direct sequencing of the *hat-Tag1* loci across a larger population in relation to SNP allelic frequency and transcript expression of candidate genes (Fig. 8).

A traditional breeding approach for altering SD is supported by our broad genetic analyses, with the genotype explaining 39% of SD phenotypic variance and 85% of the variance when considering only extreme SD accessions [54]. The strong genotypic effect is further corroborated by the genomic heritability (${h}^2$), averaging 0.61 across datasets (Fig. 2A). These values align with other studies that show strong genotypic effects on stomatal traits, and particularly on SD, across various plant species [17, 19, 34, 57]. The observed ${h}^2$ values in apple were higher than those reported for sorghum *Sorghum bicolor* (L.), which varied from 0.43 to 0.46 [17], and comparable to rice, which ranged from 0.55 to 0.66 [19]. Furthermore, SD had a moderately high predictive ability using the genomic selection model, averaging 0.41 (Fig. 2B). This value aligns with the predictive abilities of various apple traits, as previously described by Jung *et al*. [48] who reported predictive abilities ranging from 0.30 to 0.60 for apple traits such as fruit firmness, soluble solids content, and acidity. This level of predictive ability highlights SD’s potential in genomic selection for apple breeding, enabling breeders to select for SD at an early stage of the breeding process. This might be used to accelerate the creation of new apple varieties via genome editing, or traditional breeding for desired stomatal traits and improved WUE [70–72]. Furthermore, the observed variation represented by differences in leaf morphology (Fig. 2A) are likely due to natural leaf-to-leaf variability, as well as subtle environmental differences within the canopy, such as light exposure or microclimatic conditions during leaf development. Structural factors such as branch type or branch height are unlikely to explain this variation, as previous work in the REFPOP orchard [54] showed minimal effect of these factors on SD. By incorporating SD measurements into genetic BLUPs for the GWAS analysis, we account for this random variation to improve the reliability of the detected associations.

Defining region-specific SD ideotypes will also important, because the trade-off between water conservation and carbon assimilation depends strongly on local climate characteristics and the production system [54]. In water-limited regions, cultivars with lower SD can confer clear physiological advantages: they tend to exhibit higher intrinsic WUE (iWUE) and reduced transpiration, thereby preserving limited water resources. Conversely, in well-irrigated orchards, higher SD cultivars may be more desirable, as they can facilitate greater stomatal conductance, enhance CO_2_ uptake, and ultimately support higher photosynthetic rates and fruit yield.

By carefully selecting for SD ideotypes tailored to regional conditions, breeders can leverage the trade-offs in physiological performance to improve both resilience and productivity. Especially in irrigated systems like apple orchards, low-SD cultivars may still be advantageous when irrigation is managed to avoid reductions in photosynthetic activity, enabling lower water use without penalizing carbon gain and productivity.

In conclusion, our study highlights a model of genetic controls associated with SD in apple. The putative candidate genes *EPF1* and *STOMAGEN* may contribute to population-wide variation in SD. The novel discovery of *CYP77A4* as a candidate gene, which may affect cell polarization and developmental progression in the stomatal lineage, offers a compelling target for functional validation. The large genotypic effect on SD; as evidenced from the phenotypic variance decomposition, genomic heritability, and predictive ability; and the important physiological impact of SD on WUE [54] highlight the potential for incorporation of SD targets in apple breeding programs. Enhancing crop resilience to climatic extremes is a fundamental challenge in the coming decades. This study provides a genetic roadmap for using stomatal development to address this challenge.

## Material and methods

### Study site and plant material

The plant material consisted of the apple REFPOP, as previously described by Jung *et al*. [53]. Our study was conducted in years 2019, 2020, and 2021 in the apple REFPOP orchard (47.2211°N, 8.6669°E) in Waedenswil, Switzerland. The orchard was planted in 2016 and located ~25 km south of the city of Zurich at ~447 m above sea level. The apple REFPOP was composed of 269 accessions representing the apple’s broad-ranging genetic diversity [53]. The 269 accessions were grown using standard integrated plant protection methods of Switzerland.

### Apple REFPOP genotyping

Genotypic data used in this study were the same as used by Jung *et al*. [53]. To summarize, high-density genome-wide SNP marker collection was provided with the plant material and resulted from the Affymetrix Axiom® Apple480K SNP genotyping array (Thermo Fisher Scientific, Waltham, MA, USA) [45]. The datasets from the SNP arrays were combined and missing data were imputed using Beagle 4.0 incorporating pedigree information [73, 74]. A collection of 303 148 biallelic SNPs was obtained after nonpolymorphic markers were eliminated. SNP positions were assigned using the doubled haploid GDDH13 (v.1.1) apple reference genome [39].

### Stomatal density phenotyping

SD analysis and quantification were performed as previously described by Zuffa *et al*. [54]. Briefly, four mature sun-exposed leaves were sampled from four distinct primary branches in October 2019, 2020, and 2021 for one replicate of each accession. Leaves were sampled from southern facing side of the tree whenever possible, always selecting branches located at mid-height of the tree and leaves at mid-length of the branch. Previous sampling conducted on the control accessions ‘Golden Delicious’, ‘Gala’, and ‘CIVG198’ (‘Modì’) within the apple REFPOP indicated that branch type and canopy position did not influence gas-exchange rates nor the SD of the sun-exposed leaves [54]. Clear nail polish (Wild Shine Nail Color, Protective base coat, Wet n Wild®, Los Angeles, California, USA) was applied in two layers to the abaxial surface of the leaves between major veins, then removed with Eco&Clear tape (Tesa SE, Norderstedt, Germany). Bright-field microscopy (DM 2500 upright microscope with a mounted DFC 7000 T camera, Leica Microsystems, Wetzlar, Germany) captured three images per imprint (872 × 654 μm) at nonadjacent locations (top, middle, and bottom) on the imprint to account for leaf variability. Approximately 3228 images (269 accessions × 4 leaves × 3 images) were taken in 2019 and 2021, while 4304 images (269 accessions × 4 leaves × 4 images) were taken in 2020.

Manual counting of stomata using ImageJ software [75] was compared with automated counting by an image detection Mask R-CNN model for automated stomatal counting. Manually counted annotated images were used to train a Mask R-CNN to identify stomatal pixels within each input image. An adaptation of the Mask R-CNN image segmentation model [76] was trained using the implementation of the ‘tensorpack’ package (v0.11) from https://github.com/tensorpack/tensorpack/tree/master/examples/FasterRCNN. The ResNet50 (v2) backbone combined with a feature pyramid network pretrained with the COCO dataset was used [77]. After adapting the architecture to identify custom categories (stomata or background), this model was fine-tuned by training it with an adaptive learning rate and default parameters with 600 of the manually counted images for 250 epochs. The resulting model was used to automatically count SD. Counting accuracy was assessed by calculating the Pearson correlation coefficient (*r*) and root mean squared error (*RMSE*) between manually counted SD and the automatically counted SD derived from the model segmentation.

From the 8815 total available images in year 2019 and 2020, a total of 6234 images were manually counted and used to generate the dataset named ‘Manual’. The manual SD counting was performed for two images for each leaf, and when the difference between the first two manually counted images was >15, a third image was also counted. The same set of images counted manually (*n_image_* = 6234) was also counted using the automated image detection Mask R-CNN model. This dataset was named ‘Automated’. Three datasets were then created: (i) the Manual dataset (*n_image_* = 6234); (ii) the Automated dataset (*n_image_* = 6234), and (iii) the Automated Fullset (*n_image_* = 12612) (Table S5). The complete set of 12612 stomatal microscope images and the Manual dataset images dataset (*n_image_* = 6234) is available at the following DOI: 10.6019/S-BIAD2028. The normality of the SD distribution in each year (2019, 2020, 2021) for each dataset was assessed using the Shapiro–Wilk test [‘shapiro.test’ function from the R package stats (v.3.6.2)]. The nonparametric Wilcoxon test with the function ‘wilcox_test’ from the R package rstatix (v.0.7.2) was applied to assess pairwise differences in SD means both across different years within each dataset and between datasets.

### Stomatal density phenotypic variance decomposition

The three different datasets: (i) the Manual dataset, (ii) the Automated dataset, and (iii) the Automated Fullset, were analyzed separately using an LMM with the function ‘lmer’ from the lme4 R package (v.1.1-35.3) [78] fitted by restricted maximum likelihood. As previously described by Zuffa *et al*. [54], genotype (i.e. accession), genotype by year interaction, and genotype by year by leaf interaction (with leaf defined as a combined factor of genotype, year, and leaf number) were assigned as random effects in the LMM to consider differences between accessions and their interaction with seasonal and sampling conditions across years, while the year was treated as a fixed effect. In the model, the leaf effect was treated as a random factor to account for within-genotype variability derived from the sampling of multiple leaves per genotype and reduce the random error. The percentage of the phenotypic variance explained by the effects of genotype, genotype by year interaction, and genotype by year by leaf interaction were extracted from the LMM fit using the function ‘VarCorr’ from the lme4 R package. The phenotypic variance linked to the fixed effects of year was calculated as the variance of the values predicted from the LMM fit when all random effects were set to zero. BLUPs of the random effects of genotype were extracted by applying the function ‘ranef’ from the lme4 R package to the LMM fit.

### Stomatal density genomic heritability and predictive ability

The BayesCπ model, implemented using the BGLR R package (v.1.1.2) [79], was used as previously described by Lehermeier *et al*. [80] to calculate the genomic variance and genomic heritability for each dataset. Genomic heritability (${h}^2$) was defined using the variance explained by the SNP markers (*V_g_*) and variance unexplained by the SNP markers (*V_e_*) as ${h}^2=\frac{V_g}{\left({V}_g+{V}_e\right)}$. The mean genomic heritability was determined using the marker effects saved in each iteration of the Gibbs sampler and averaged over iterations.

The genomic prediction model genomic BLUP (G-BLUP) was applied as previously described by Jung *et al*. [48]. This model was based on the genomic relationship matrix **G**, which was derived from the cross-product of centered and standardized genomic matrix **M** (*n* x *m* matrix with *n* = 269 genotypes and *m* = 303 148 SNP markers), divided by the total number of SNPs *m* [81]. The model is expressed as:


(1) \begin{equation*} y=1\mu +u+\varepsilon \end{equation*}

where *y* represented the response vector of BLUPs for one of the SD datasets, *u* was a vector of random effects following $u\sim N\left(0,G{\sigma}_u^2\right)$with variance ${\sigma}_u^2$, and *ɛ* was a vector of residuals $\varepsilon \sim N\left(0,I{\sigma}_{\varepsilon}^2\right)$ with identity matrix *I* and error variance ${\sigma}_{\varepsilon}^2$. The G-BLUP model was implemented using a semi-parametric Bayesian reproducing kernel Hilbert spaces regression algorithm within the R package BGLR (v.1.1.2). Specifically, the G-BLUP was applied with 12000 iterations of the Gibbs sampler, a thinning of five, and a burn-in of 2000 discarded samples. For each of the SD datasets separately, a 10-fold cross-validation was performed and repeated 10 times. The 269 apple REFPOP accessions were split randomly into folds without replacement, resulting in 100 model runs for each SD dataset. Each model run used 90% of the total number of accessions as the training set and 10% as the validation set. Predictive ability was assessed with the Pearson correlation coefficient between BLUPs and predicted values for all the validation sets (yielding 100 values of predictive ability per SD dataset). The predictive ability of a genomic selection model is defined as the correlation between the phenotypes and the genomic estimated breeding values produced by the genomic prediction model [82].

### Stomatal density genome-wide association study

To identify marker-trait associations, a GWAS was performed for each of the three datasets using the SD genetic BLUPs and the genomic matrix **M***,* as defined in the previous paragraph. Two types of GWAS models were applied: (i) the Bayesian-information and linkage disequilibrium iteratively nested keyway (BLINK) [83] implemented in the R package GAPIT (v.3.0) [84], and (ii) the genome-wide efficient mixed model analysis for association studies (GEMMA) [47]. GEMMA is an exact statistical single-locus method that tests the association between each single locus and the trait of interest, one locus at a time, and uses all available SNPs to estimate kinship matrix. BLINK is a multilocus approximation of the exact statistical methods with an optimized fixed-effect model especially suitable for detecting associations with closely linked loci [50, 83].

This study applied a resampling approach to GWAS, which was inspired by the first step of the *de novo* GWAS algorithm [85]. Ten subsets made of 90% of accessions each were drawn from the whole dataset consisting of 269 accessions. During the sampling of each subset, 10% of the 269 accessions were randomly sampled without replacement and excluded from the subset. The same sampling of accession subsets was repeated ten times (for 10 different seeds defined by the R function ‘set.seed’), thus creating 100 different subsets, with each containing 90% of the accessions. Using these subsets, a total of 100 GWASs were then performed using each GWAS model (BLINK and GEMMA) and for each of the three different datasets (Manual, Automated, and Automated Fullset). Weak population structure was described for the analyzed 269 apple accessions [53]. Therefore, the GWAS models were applied with no principal components used as covariates. SNPs with a minor allele frequency lower than 0.05 were removed from the analysis. For GEMMA, the threshold for SNP selection was set at a −log_10_(*P*-value) of 5, as GEMMA showed lower power to detect significant associations than BLINK. For BLINK, marker–trait associations were considered significant for *P-*values lower than a Bonferroni-corrected significance level α = α/*m* with α = 0.05 (−log_10_(*P*) > 6.78), which is named here as the ‘standard Bonferroni threshold’. An additional Bonferroni-corrected significance level adjusted for the number of GWASs was also applied, named as the ‘adjusted Bonferroni threshold’, where *α* = *α*/(*m × g*) (−log_10_(*P*) > 8.78) with *g* = 100 indicating the number of GWAS for each model and dataset.

Significant marker–trait associations from the 600 GWAS were compared in Manhattan plots. The coefficient of determination (${R}^2$) was used to indicate the proportion of phenotypic variance explained by each significantly associated SNP. The ${R}^2$ was measured with a linear regression model which was fitted with the BLUPs as the response variable and a vector of SNP marker values (coded as 1, 2, and 3 for allele dosage) as predictor [48].

### Stomatal density candidate gene identification

A robust association is defined in this work as being characterized by high ${R}^2$ values, significant low *P*-values, and consistently identified by Manual and Automated datasets using BLINK. To explore candidate genes in the vicinity of the robust associations, PLINK (v.1.9) [86] with its default settings was used to create haplotype blocks [87], in which the robust associations were localized. A pairwise LD heatmap was generated using the R package ‘LDheatmap’ (v1.0-6) [88] for genomic regions containing the robust associations and the respective haploblocks. The search for candidate genes was performed based on the genes localized in the doubled haploid reference genome GDDH13 (v1.1) [39]. Candidate genes were identified based on their physiologically and biologically linked roles inferred from putative orthologues in *A. thaliana* using TAIR (www.arabidopsis.org) and localized in the genome browser for apple (www.rosaceae.org).

### Gene co-expression network analysis and functional enrichment analysis of stomatal density

To use identified candidate genes as reference points for identifying relevant co-expression networks and to explore gene expression, a weighted gene co-expression network analysis (WGCNA) (v.72-5) [89, 90] and Cytoscape (v.3.07) [91] of differentially expressed genes was generated using 150 apple RNA sequencing data publicly available in the National Center for Biotechnology Information (https://www.ncbi.nlm.nih.gov/sra, Table S2) ([92–94], 2021; [95–100]). These data included various plant experiments, involving different plant tissues: 6 flower buds, 9 opened flowers, 9 anthers, 1 filament, 9 stigma, 1 style, 9 ovaries, 1 petal, 1 pollen, 1 receptacle, 1 sepal, 55 leaves, 30 roots, 27 fruits, and 6 fruit skins. The correlation matrix was filtered to retain only correlations with absolute values > 0.6 tpm (transcripts per million) (*|r| > 0.6*) and Bonferroni-corrected values with *P ≤ 0.05*. Furthermore, a functional singular enrichment analysis was performed at AgriGO (v.2.0) [101], using the doubled haploid reference genome GDDH13 (v.1.1) [39]. To minimize the false discovery rate (FDR), Benjamini–Hochberg correction [102] was used to adjust the original *P*-values. Differentially expressed genes were defined when an associated adjusted *P_FDR_* ≤ 0.05 was observed.

To identify the co-expression modules containing *EPF1* and *STOMAGEN*, we screened these genes across the 69 gene co-expression networks (modules) generated by the WGCNA. Both genes were located in network 11, which comprised 647 nodes (genes) and 4289 edges (connections). For downstream analyses, we extracted only the first-neighboring genes of our identified candidates (*STOMAGEN* and *EPF1*) as well as of other known stomatal regulatory genes (*EPF2*, *FAMA*, *SCREAM*, and the paralog *STOMAGEN*). The resulting gene correlation network consisted of 79 genes that were significantly co-expressed (*P* < 0.001). This refined subnetwork was subsequently used to identify a novel putative candidate gene involved in stomatal regulation, CYTOCHROME P450 (*CYP77A4*).

### Whole genome sequencing of apple accessions

Fourteen apple accessions from the apple REFPOP were selected for whole genome sequencing, as described by Watts *et al*. [103]. Although the number of genome assemblies available was limited (*n* = 14), a targeted analysis was performed to examine structural variation in the regions surrounding the identified candidate genes.

The selected accessions were chosen based on the following criteria: (i) phenotypic extremes to represent the full range of SD and accessions with high and low values included, as previously described by Zuffa *et al*. [54]; (ii) genetic diversity to include a wide range of genetic backgrounds within the apple REFPOP. Accessions were selected for historical reasons (‘Granny Smith’), relatedness (‘Esopus Spitzenburg’ is a parent of ‘Jonathan’), and haplotype diversity (‘Fiessers Erstling’ and ‘Tropical Beauty’), thereby maximizing the phenotypical and genotypical diversity in SD and exploiting the SD subset previously analyzed by [54]; and (iii) historical and commercial significance to ensure the inclusion of cultivars with notable historical or market relevance.

To extract high-molecular-weight DNA for whole genome sequencing, fresh healthy leaf tissue was collected from the 14 apple accessions and immediately flash-frozen in liquid nitrogen. Each sample was collected from a single tree for each accession in 2023. DNA extraction, library preparation, and sequencing were carried out by the Arizona Genomics Institute (USA), targeting ~30× genome coverage using the Revio system (Pacific Biosciences, Menlo Park, CA, USA) with HiFi long-read technology. High-molecular-weight DNA was extracted from the collected leaves using the modified cetyltrimethylammonium bromide (CTAB) protocol [104]. Frozen DNA purity was measured with Nanodrop (Thermo Fisher Scientific, Waltham, MA, USA), DNA concentration was measured with Qubit HS kit (Invitrogen, Waltham, MA, USA).

The high-molecular-weight DNA was sized by Femto Pulse System (Agilent, Santa Clara, CA, USA) and ~10 μg of DNA were sheared to specific size range (15–20 kb) using Megaruptor 3 (Diagenode, Denville, NJ, USA). The PacBio HiFi (PacBio, Menlo Park, CA, USA) sequencing library was constructed using SMRTbell Express Template Prep Kit 2.0. The final library was size-selected on a Pippin HT (Sage Science, Beverly, MA, USA) using an S1 marker with a size selection at the 10 to −25 kb range. This final library was quantified with Qubit HS kit (Invitrogen, Waltham, MA, USA) and size-checked on Femto Pulse System (Agilent, Santa Clara, CA, USA). The sequencing library was sequenced on a Sequel II instrument (PacBio, Menlo Park, CA, USA) on 8 M SMRT® Cells in CCS mode for 30 hours.

### Genome assemblies for detection of structural variation in apple

Whole genome sequencing is described by Watts *et al*. [103] and briefly reviewed here: haplotype-resolved genome assemblies, with an average length of 675.3 Mb and 47 445 annotated protein-coding genes, provide a high level of completeness (mean BUSCO score of 98.8%) and capture novel genetic diversity. These assemblies identify 578 uncharacterized orthogroups. To extract homologous genomic regions across the sequenced genomes, all positions were anchored using the doubled haploid reference genome GDDH13 (v.1.1) [39], from which start and end points on the chromosomes were determined per quantitative trait locus (QTL). A pan-genome graph was constructed, using ODGI [105], with all homologous chromosomes. Homologous regions were extracted in ‘fasta’ format using the ODGI extract command with the option ‘full-range’. To identify transposable elements, a BLAST database was constructed using transposable elements identified by Luo *et al*. [106] based on the apple genome described by Velasco *et al*. [42], using ‘makeblastdb’. The homologous chromosome regions were searched against this database using ‘blastn’ [42] with the option ‘-outfmt 6’. To identify homologous genes, the positions of annotated genes (from the GDDH13 gff3 file) were parsed using *awk* to create a ‘bed’ file. The corresponding regions were extracted in ‘fasta’ format using ‘bedtool getfastav’ with the ‘-fov’ option. These gene sequences were used to construct a nucleotide BLAST database (‘makeblastdb’), which the homologous chromosome regions were searched against using ‘blastn’ with the options ‘-outfmt 6 -evalue 1e-10’. The resulting BLAST output was then loaded into R and redundant transposable elements were excluded if their start and end were greater and lower, respectively, than any other BLAST hit. All duplicated records were removed using the ‘duplicated’ function. All figures in this manuscript were created using the R package ggplot2 (v4.2.1) [107], and postprocessing in Affinity Publisher 2 (v2.3.0) (Serif, Nottingham, UK).

## Supplementary Material

Web_Material_uhag285

## Data Availability

SNP genotypic data are available at https://doi.org/10.15454/IOPGYF. Genome sequence data and assemblies are available at NCBI under BioProject PRJNA1168485. Images used to quantify stomatal density will be available at 10.6019/S-BIAD2028. Any data supporting the results of this study not published in the manuscript can be found in the Supplementary Information.
