# Supplementary material for: Candidate genes with antagonistic roles in stomatal development are associated with population-wide variation in apple
Source: Hortic Res. 2026 Jul 8;13(10):uhag285. doi: 10.1093/hr/uhag285 (PMC13634675; doi:10.1093/hr/uhag285)
Supplement: Supplementary file 1 [file web_material_uhag285.zip › Figure_S1.pdf]

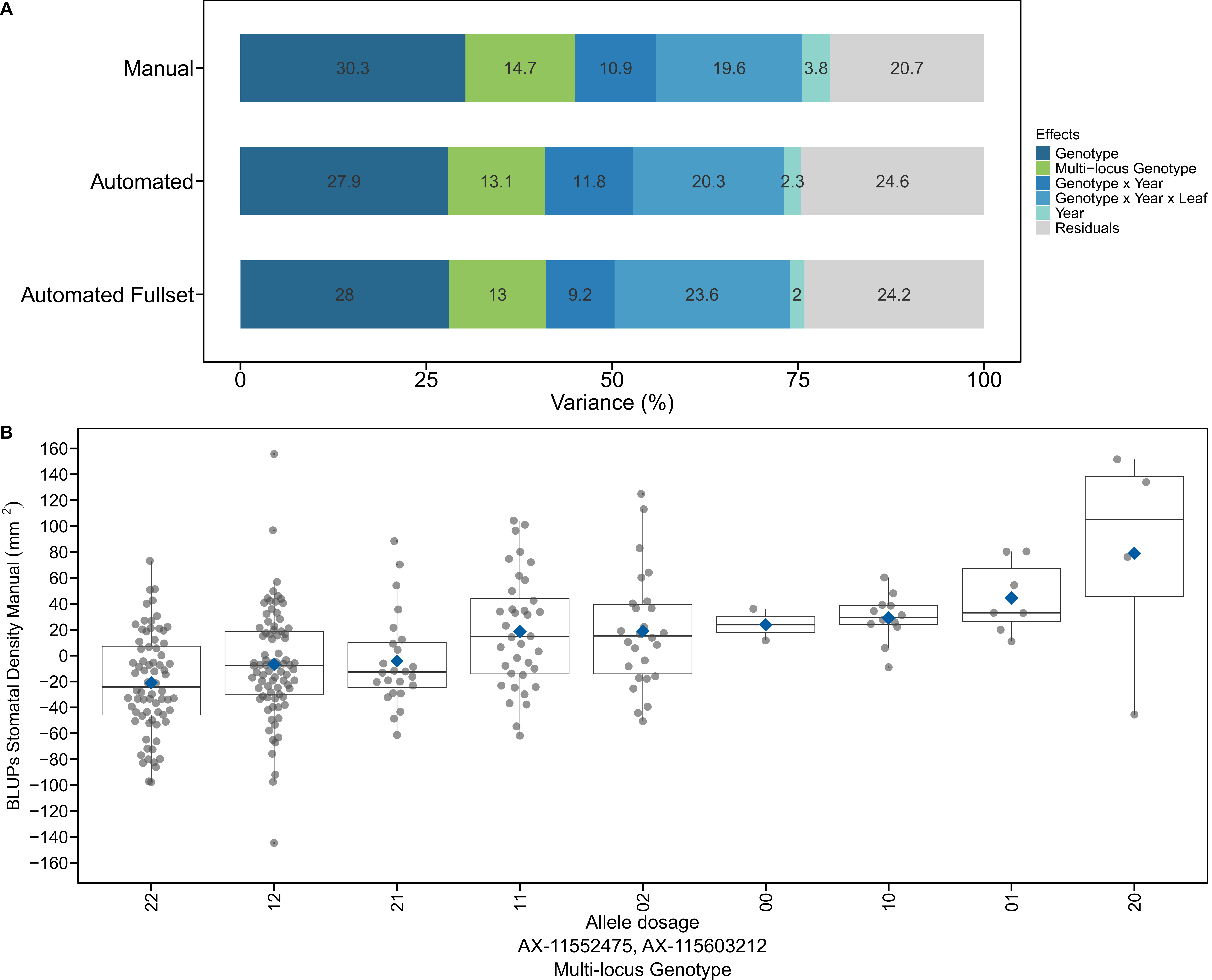
A
Manual
Automated
Automated Fullset
30.3
14.7
10.9
19.6
3.8
20.7
27.9
13.1
11.8
20.3
2.3
24.6
28
13
9.2
23.6
2
24.2
0
25
50
75
100
Variance (%)
Effects
Genotype
Multi−locus Genotype
Genotype x Year
Genotype x Year x Leaf
Year
Residuals
B
BLUPs Stomatal Density Manual (mm 2)
160
140
120
100
80
60
40
20
0
-20
-40
-60
-80
−100
−120
−140
−160
22
12
21
11
02
00
10
01
20
Allele dosage
AX-11552475, AX-115603212
Multi-locus Genotype
