# Supplementary material for: Candidate genes with antagonistic roles in stomatal development are associated with population-wide variation in apple
Source: Hortic Res. 2026 Jul 8;13(10):uhag285. doi: 10.1093/hr/uhag285 (PMC13634675; doi:10.1093/hr/uhag285)
Supplement: Supplementary file 1 [file web_material_uhag285.zip › Figure_S2.pdf]

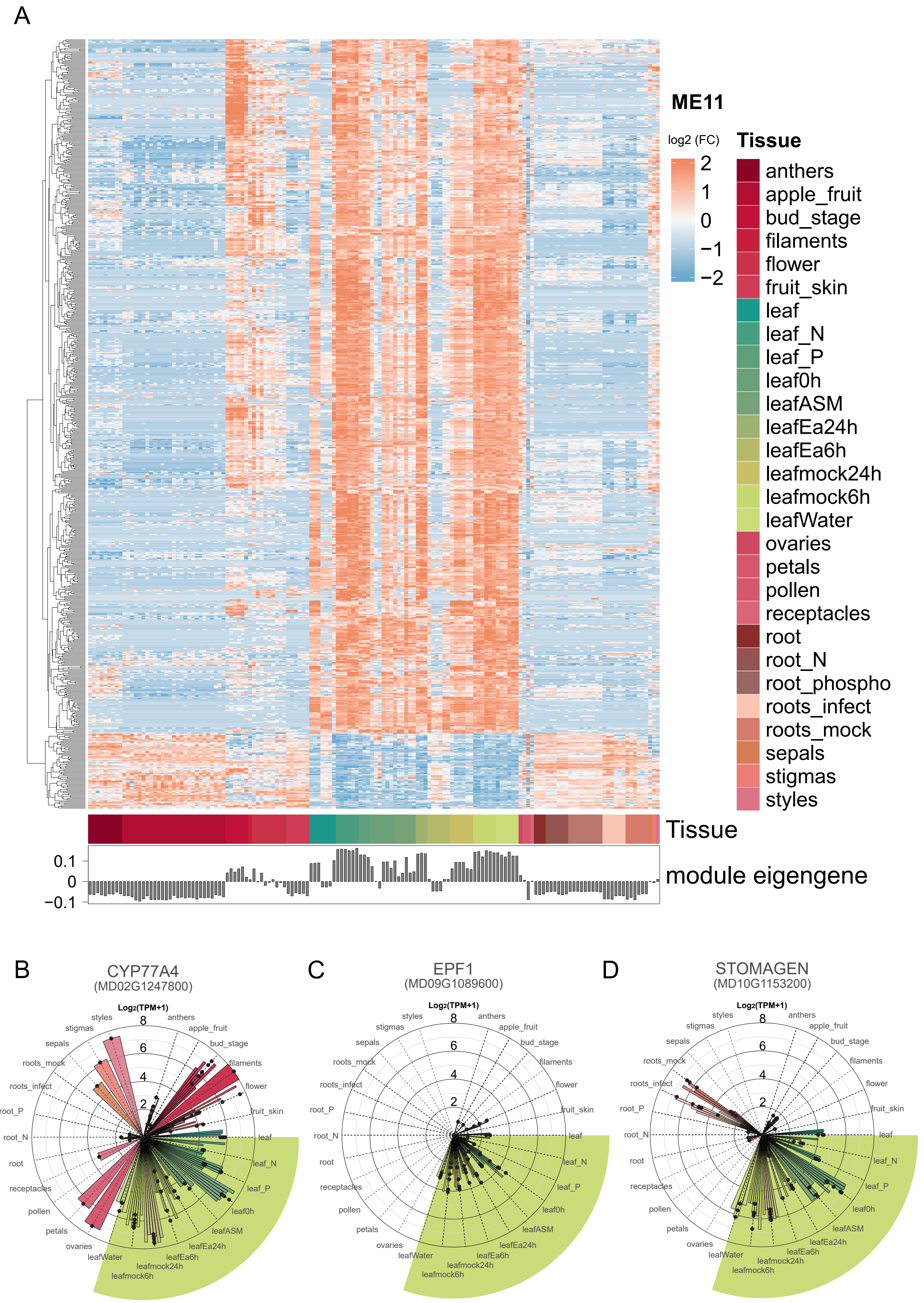
A
ME11
log2 (FC)
2
1
0
−1
−2
Tissue
anthers
apple_fruit
bud_stage
filaments
flower
fruit_skin
leaf
leaf_N
leaf_P
leaf0h
leafASM
leafEa24h
leafEa6h
leafmock24h
leafmock6h
leafWater
ovaries
petals
pollen
receptacles
root
root_N
root_phospho
roots_infect
roots_mock
sepals
stigmas
styles
Tissue
module eigengene
0.1
0
−0.1
B
CYP77A4
(MD02G1247800)
C
EPF1
(MD09G1089600)
D
STOMAGEN
(MD10G1153200)
Log2(TPM+1)
8
6
4
2
styles
anthers
stigmas
apple_fruit
sepals
bud_stage
roots_mock
filaments
roots_infect
flower
root_P
fruit_skin
root_N
leaf
root
leaf_N
receptacles
leaf_P
pollen
leaf0h
petals
leafASM
ovaries
leafEa24h
leafWater
leafEa6h
leafmock24h
leafmock6h
