# Supplementary material for: Candidate genes with antagonistic roles in stomatal development are associated with population-wide variation in apple
Source: Hortic Res. 2026 Jul 8;13(10):uhag285. doi: 10.1093/hr/uhag285 (PMC13634675; doi:10.1093/hr/uhag285)
Supplement: Supplementary file 1 [file web_material_uhag285.zip › Figure_S3.pdf]

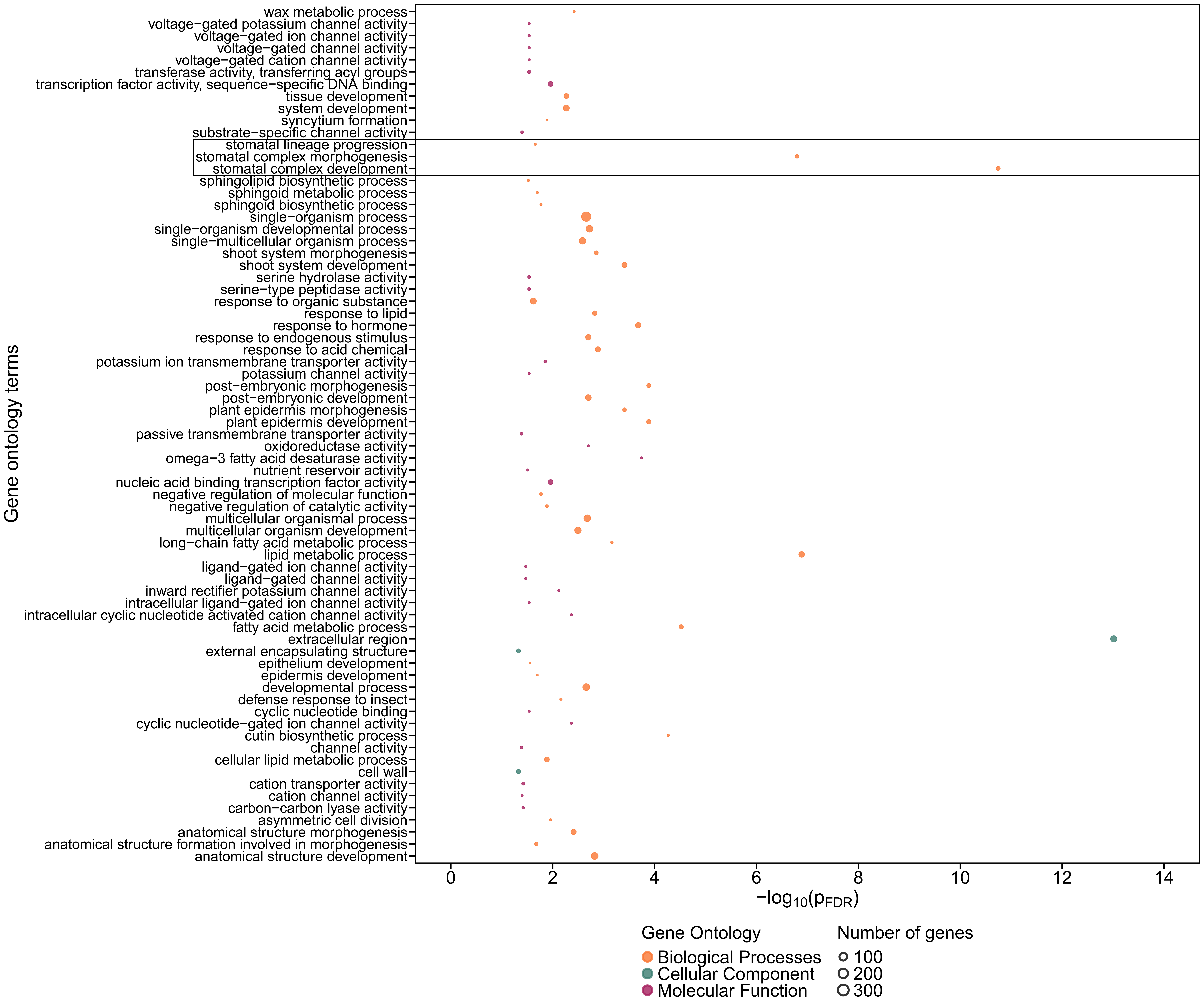
wax metabolic process
voltage−gated potassium channel activity
voltage−gated ion channel activity
voltage−gated channel activity
voltage−gated cation channel activity
transferase activity, transferring acyl groups
transcription factor activity, sequence−specific DNA binding
tissue development
system development
syncytium formation
substrate−specific channel activity
stomatal lineage progression
stomatal complex morphogenesis
stomatal complex development
sphingolipid biosynthetic process
sphingoid metabolic process
sphingoid biosynthetic process
single−organism process
single−organism developmental process
single−multicellular organism process
shoot system morphogenesis
shoot system development
serine hydrolase activity
serine−type peptidase activity
response to organic substance
response to lipid
response to hormone
response to endogenous stimulus
response to acid chemical
potassium ion transmembrane transporter activity
potassium channel activity
post−embryonic morphogenesis
post−embryonic development
plant epidermis morphogenesis
plant epidermis development
passive transmembrane transporter activity
oxidoreductase activity
omega−3 fatty acid desaturase activity
nutrient reservoir activity
nucleic acid binding transcription factor activity
negative regulation of molecular function
negative regulation of catalytic activity
multicellular organismal process
multicellular organism development
long−chain fatty acid metabolic process
lipid metabolic process
ligand−gated ion channel activity
ligand−gated channel activity
inward rectifier potassium channel activity
intracellular ligand−gated ion channel activity
intracellular cyclic nucleotide activated cation channel activity
fatty acid metabolic process
extracellular region
external encapsulating structure
epithelium development
epidermis development
developmental process
defense response to insect
cyclic nucleotide binding
cyclic nucleotide−gated ion channel activity
cutin biosynthetic process
channel activity
cellular lipid metabolic process
cell wall
cation transporter activity
cation channel activity
carbon−carbon lyase activity
asymmetric cell division
anatomical structure morphogenesis
anatomical structure formation involved in morphogenesis
anatomical structure development
Gene ontology terms
0
2
4
6
8
10
12
14
$-\log_{10}(p_{FDR})$
Gene Ontology
Biological Processes
Cellular Component
Molecular Function
Number of genes
100
200
300
