# Supplementary material for: Candidate genes with antagonistic roles in stomatal development are associated with population-wide variation in apple
Source: Hortic Res. 2026 Jul 8;13(10):uhag285. doi: 10.1093/hr/uhag285 (PMC13634675; doi:10.1093/hr/uhag285)
Supplement: Supplementary file 1 [file web_material_uhag285.zip › Figure_S4.pdf]

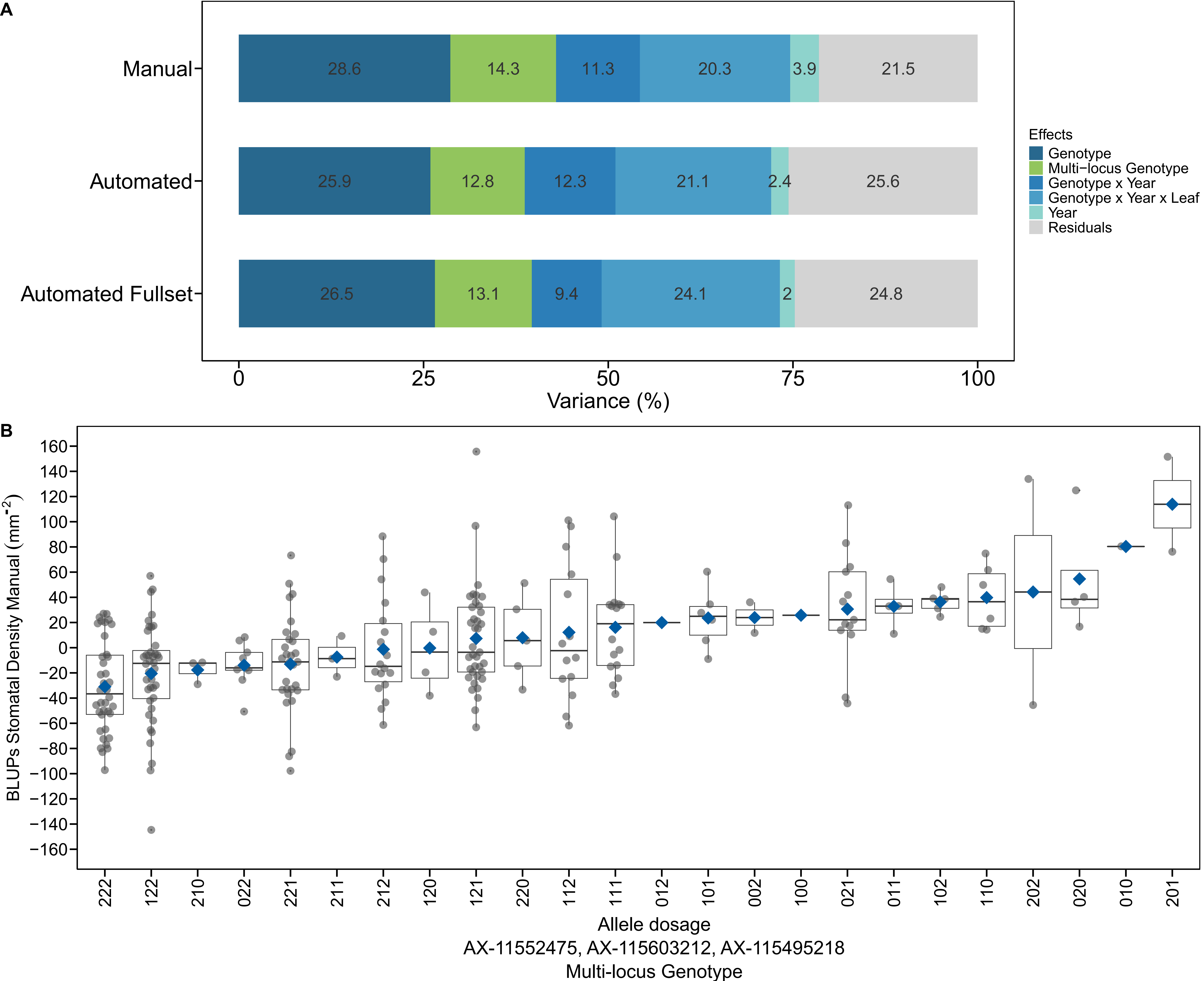
A
Manual
Automated
Automated Fullset
28.6
14.3
11.3
20.3
3.9
21.5
25.9
12.8
12.3
21.1
2.4
25.6
26.5
13.1
9.4
24.1
2
24.8
0
25
50
75
100
Variance (%)
Effects
Genotype
Multi−locus Genotype
Genotype x Year
Genotype x Year x Leaf
Year
Residuals
B
BLUPs Stomatal Density Manual (mm-2)
160
140
120
100
80
60
40
20
0
−20
−40
−60
−80
−100
−120
−140
−160
222
122
210
022
221
211
212
120
121
220
112
111
012
101
002
100
021
011
102
110
202
020
010
201
Allele dosage
AX-11552475, AX-115603212, AX-115495218
Multi-locus Genotype
